# SMAD3/SP1 complex‐mediated constitutive active loop between lncRNA PCAT7 and TGF‐β signaling promotes prostate cancer bone metastasis

**DOI:** 10.1002/1878-0261.12634

**Published:** 2020-02-08

**Authors:** Chuandong Lang, Yuhu Dai, Zhengquan Wu, Qing Yang, Shaofu He, Xin Zhang, Wei Guo, Yingrong Lai, Hong Du, Hehe Wang, Dong Ren, Xinsheng Peng

**Affiliations:** ^1^ Department of Orthopaedic Surgery The First Affiliated Hospital Sun Yat‐sen University Guangzhou China; ^2^ Guangdong Provincial Key Laboratory of Orthopedics and Traumatology Guangzhou China; ^3^ Department of Radiology The First Affiliated Hospital Sun Yat‐sen University Guangzhou China; ^4^ Clinical Experimental Center Jiangmen Central Hospital Affiliated Jiangmen Hospital Sun Yat‐sen University Jiangmen China; ^5^ Department of Pathology The First Affiliated Hospital Sun Yat‐sen University Guangzhou China; ^6^ Department of Pathology The First People’s Hospital of Guangzhou City Guangzhou China; ^7^ Department of Medical Laboratory Weifang Medical University Weifang China

**Keywords:** bone metastasis, lncRNA PCAT7, miR‐324‐5p, prostate cancer, TGF‐β signaling

## Abstract

Bone metastasis is associated with cancer‐related death in patients with prostate cancer (PCa). Long noncoding RNAs (lncRNAs) play critical roles in tumor progression of PCa. Nevertheless, the biological function of lncRNAs in PCa bone metastasis remains unclear. PCAT7 was identified as a bone metastasis‐related lncRNA via analyzing TCGA dataset. Meanwhile, PCAT7 was found to be elevated in primary PCa tissues with bone metastasis and associated with bone metastasis status and poor prognosis of patients with PCa. Functionally, our results reveal that PCAT7 overexpression promotes PCa bone metastasis *in vivo*, as well as migration, invasion, and EMT of PCa cells *in vitro*; on the contrary, PCAT7 knockdown has an inverse effect. Mechanistically, PCAT7 activates TGF‐β/SMAD signaling by upregulating TGFBR1 expression via sponging miR‐324‐5p. In turn, TGF‐β signaling forms a positive feedback loop with PCAT7 via SMAD3/SP1 complex‐induced PCAT7 upregulation. Finally, the clinical positive correlation between PCAT7 and TGFBR1 and TGF‐β signaling activity, and the negative association with miR‐324‐5p are further demonstrated in PCa tissues and clinical primary PCa cells. This study reveals a novel mechanism that is responsible for the constitutive activation of TGF‐β signaling in PCa bone metastasis, implying that PCAT7 can act as a potential therapeutic target against bone metastasis of PCa via disrupting the constitutive active loop between PCAT7 and TGF‐β signaling.

## Graphical abstract

lncRNA PCAT7 is upregulated in primary prostate cancer tissues with bone metastasis. PCAT7 activates TGF‐β/SMAD signaling by upregulating TGFBR1 expression via sponging miR‐324‐5p. In turn, TGF‐β signaling forms a positive feedback loop with PCAT7 via SMAD3/SP1 complex‐induced PCAT7 upregulation. This feedback loop promotes prostate cancer bone metastasis.

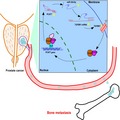

**Abbreviations**
EMT: epithelial–mesenchymal transition
H&E: hematoxylin and eosin stain
miR‐324‐5p: microRNA‐324‐5p
MMP13: matrix metallopeptidase 13
PCAT7: prostate cancer‐associated transcript 7
PCR: polymerase chain reaction
SMAD2/3/4: SMAD family member 2/3/4
SP1: Sp1 transcription factor
TCGA: The Cancer Genome Atlas
TGFBR1: transforming growth factor beta receptor 1

## 1 Introduction

Prostate cancer (PCa) is the second leading cause of cancer‐related death among men in the United States (Siegel *et al.*, 2017, 2018). Its high mortality rate is mainly due to metastases to distinct organs, including bone (Gartrell *et al.*, 2015). The skeleton is the most common metastatic site of PCa, and bone metastasis occurs in up to 70% of patients who die of PCa. Metastatic bone disease greatly contributes to the decline in the quality of life and final death, due to treatment and skeletal complications, including pain, compression of the spinal cord, pathological fractures, hypercalcemia, and spinal instability (Coleman, 2006). Current treatments for bone metastasis are not curative and can only relieve symptoms but often fail to prevent its progression (Macedo *et al.*, 2017). Hence, it is urgent for revealing the molecular mechanism underlying bone metastasis of PCa, in order to develop new therapeutic strategies.

Long noncoding RNAs (lncRNAs) are a class of RNA transcripts with no protein‐coding capacity that is greater than 200 nt in length. They regulate many biological processes through various mechanisms, including scaffolds or guides to regulate interactions between proteins and genes, decoys to bind proteins, and enhancers to modulate transcription of their target genes (Chen *et al.*, 2018). Notably, recent studies have identified that lncRNAs can function as competitive endogenous RNAs (ceRNA) and are able to serve as a ‘miRNA sponge’ to derepress miRNA‐targeted mRNA expression (Karreth *et al.*, 2011; Tay *et al.*, 2011). Increasing evidence has indicated that lncRNAs play an important role in the metastasis of human cancers, including PCa (Li *et al.*, 2017a; Lin and Yang, 2018; Prensner *et al.*, 2011; Prensner *et al.*, 2013; Zhou *et al.*, 2018). For instance, the crosstalk between ROR1–HER3 and the Hippo–YAP pathway promotes bone metastasis of breast cancer in a lncRNA‐dependent manner (Li *et al.*, 2017a). Nevertheless, up to now, the biological functions of lncRNAs in bone metastasis of PCa are little known.

Emerging studies found that the dysregulation of lncRNAs contributes to the metastasis of cancers via multiple signaling pathways (Li *et al.*, 2017a; Yue *et al.*, 2018; Zhang *et al.*, 2018). Multiple signaling pathways, including the NF‐κB, Notch, Wnt, and TGF‐β, have been well documented to be participated in bone metastasis of PCa (Fournier *et al.*, 2015; Li *et al.*, 2017b; Ren *et al.*, 2017; Zayzafoon *et al.*, 2004). Among these, TGF‐β has been reported to be the crucial contributor to bone metastasis of PCa via inducing EMT or promoting the invasiveness of cancer cells (Tan *et al.*, 2012; Yu *et al.*, 2016). Since its discovery, TGF‐β pathway has been found to take part in many cellular processes, such as differentiation, migration, and apoptosis (Shi and Massague, 2003), and its dysregulation is associated with heritable and vascular disorders, fibrosis, and cancer (Massague *et al.*, 2000). TGF‐β signaling plays a dual role in tumor progression of human cancers. In early tumor initiation, TGF‐β signaling suppresses the formation and progression of human cancers. Conversely, TGF‐β functions as an oncogenic signal during the late stages of cancer (Ying *et al.*, 2017). In bone metastasis of cancer, cancer cells that metastasize to the bone are driven by TGF‐β signaling, which can result in an abnormal bone remodeling process. Reciprocally, bone microenvironment promotes the proliferation of cancer cells through osteoblast‐producing growth factors, including TGF‐β (Weilbaecher *et al.*, 2011), which leads to a vicious cycle, in which TGF‐β is a significant component. Indeed, TGF‐β signaling has been widely reported to promote bone metastasis of multiple human cancers, including PCa and breast cancer (Dai *et al.*, 2017; Fournier *et al.*, 2015; Kang *et al.*, 2005; Korpal *et al.*, 2009; Yin *et al.*, 1999). Importantly, therapies targeting TGF‐β present favorable benefits for antibone metastasis treatment of cancer (Hu *et al.*, 2012; Wan *et al.*, 2012). Therefore, identification of critical functional molecules that disrupt the constitutive active loop of TGF‐β signaling will accelerate the development of strategies against bone metastasis of cancer.

In this study, we identified a PCa‐associated lncRNA, prostate cancer‐associated transcript 7 (PCAT7), also known as PCAN‐R2, which locates in chromosome (chr) 9q22.32 and has been reported to be involved in tumor progression (Liu *et al.*, 2017b; Liu *et al.*, 2017a). The results demonstrated that PCAT7 was elevated in primary PCa tissues with bone metastasis. Further, we found that SMAD3/SP1 transcriptional complex‐induced overexpression of PCAT7 upregulated TGFBR1 expression by sponging miR‐324‐5p as a ceRNA, which led to the unrestrained activation of TGF‐β pathway, which reciprocally promoted PCa bone metastasis. Collectively, our study implies that SMAD3/SP1 complex mediates the double‐positive loop between PCAT7 and TGF‐β signaling, suggesting that PCAT7 can act as a potential therapeutic target for bone metastasis of PCa.

## 2 Materials and methods

### 2.1 Cell culture

PCa cell lines (LNCaP, PC‐3, 22RV1, VCaP, DU145) and normal prostate epithelial cells (RWPE‐1) were provided by the Shanghai Chinese Academy of Sciences Cell Bank (China). C4‐2B PCa cell line was obtained from the MD Anderson Cancer Center. Abovementioned human cells were cultured as described previously (Dai *et al.*, 2019).

### 2.2 Plasmid and transfection

The following plasmids were purchased from the GeneCopoeia Company (Guangzhou, China): SP1, PCAT7 expression vector, and short hairpin RNA (shRNA) against PCAT7. Plasmids encoding TGFBR1 and SMAD2/3/4 were obtained from the laboratory of Professor Libing Song (Sun Yat‐sen University, China). Small interfering RNA (siRNA) for the SMAD3 was purchased from RiboBio (Guangzhou, China). The target sequences were as follows: shPCAT7#1, 5′‐ACAGGAAGCTCTAGCAGTA‐3′; shPCAT7#2, 5′‐CCATCAACAGTGAGAGGAA‐3′; siSMAD3#1, 5′‐GGTGCTCCATCTCCTACTA‐3′; and siSMAD3#2, 5′‐GAGGCTGTCTACCAGTTGA‐3′. Retroviral production and infection were performed as previously described (Hahn *et al.*, 2002; Yang *et al.*, 2019).

### 2.3 Real‐time quantitative PCR (qRT‐PCR)

Total RNA was extracted from PCa tissues and cells with TRIzol reagent (Invitrogen, Carlsbad, CA, USA). Primers for U6 and miR‐324‐5p were purchased from RiboBio. The specific process of qRT‐PCR was described in our previous study (Ren *et al.*, 2017). The detailed information of primers is presented in Table S1.

### 2.4 Human samples

A total of 57 PCa tissues were collected at the First People’s Hospital of Guangzhou City (Guangzhou, China) between May 2008 and March 2018. The informed consent was obtained from all patients. The detailed information of all patients is shown in Table S2. This study was according to the Declaration of Helsinki and ethically approved by the Institutional Review Board.

### 2.5 Western blotting

Western blotting was performed as described previously (Ren *et al.*, 2013). Antibodies against p‐SMAD3, SMAD3, vimentin, fibronectin, and E‐cadherin were provided by Cell Signaling Technology, while antibodies against TGFBR1 were provided by Invitrogen. Anti‐β‐actin (Proteintech, Chicago, IL, USA) and anti‐p84 (Invitrogen) antibodies were used as the loading control.

### 2.6 Luciferase reporter assay

A total of 3 × 10^3^ cells were cultured in 48‐well plates for 1 day. The control luciferase plasmid or luciferase reporter plasmids (100 ng) were added to 1 ng of pRL‐TK Renilla plasmid (Promega, Madison, WI, USA) and transfected into cells using the Lipofectamine 3000 reagent (Invitrogen). Twenty‐four hours after transfection, luciferase and Renilla signals were measured using the Dual Luciferase Reporter Assay Kit (Promega), according to the manufacturer’s instructions.

### 2.7 RNA immunoprecipitation (RIP) and chromatin immunoprecipitation (ChIP) assays

The RIP assay was conducted using a Thermo Fisher RIP Kit (Thermo Fisher Scientific, Waltham, MA, USA) following the manufacturer’s instructions. In brief, the cells were lysed in a RIP lysis buffer, and RNA magnetic beads were conjugated with a human anti‐Ago2 antibody or with a negative control normal mouse anti‐IgG. Subsequently, the retrieved RNA was assayed using real‐time PCR**.** ChIP‐re‐ChIP was performed as previously described (Reid *et al.*, 2003). An EZ ChIP Chromatin Immunoprecipitation Kit (Millipore, Bedford, MA, USA) was used following the manufacturer’s instructions. Cross‐linked chromatin was sonicated into fragments, and then, the fragments were immunoprecipitated using SP1 or SMAD3 antibodies (Yue *et al.*, 2018). The immunoprecipitated complexes were eluted using a re‐ChIP buffer. The primers used in ChIP‐PCR assays are provided in Table S3.

### 2.8 RNA FISH and subcellular fractionation of PCAT7

Cy3‐labeled PCAT7 was obtained from RiboBio. RNA fluorescence *in situ* hybridization (FISH) was performed using a FISH Kit (RiboBio), following the manufacturer’s instructions. A nucleus and cytoplasm segmentation PARIS™ Kit (Ambion, Austin, TX, USA) was used to segment the nucleus and cytoplasm of cells following the manufacturer’s instructions.

### 2.9 Migration and invasion assays

Transwell assays were performed to determine the migration and invasion capability of PCa cells following the previous description (Dai *et al.*, 2017). Invasion and migration assays were performed using a Transwell chamber consisting of 8‐mm membrane filter inserts (Corning, NY, USA) with or without Matrigel (BD Biosciences, Franklin Lakes, NJ, USA) coating.

### 2.10 Animal study

All mouse experiments were approved by the Institutional Animal Care and Use Committee of Sun Yat‐sen University and performed as previously described (Ren *et al.*, 2017). Ten mice were used in every group for animal experiments. Luciferase plasmids were first stably transfected into the PC3 cells. Then, the luciferase‐labeled PC3 cells were injected into the left ventricle of anesthetized mice. After 10 min of cell inoculations, D‐luciferin (150 mg·kg^−1^, Caliper Life Sciences, Boston, MA, USA), the substrate of bioluminescence signal to luciferase, was intraperitoneally injected into the anesthetized mice, and bioluminescence imaging (BLI) was captured in the light‐tight box of the IVIS Imaging System (Caliper Life Sciences). The detailed procedures regarding the luciferase‐expressing PC3 cells and the detection of BLI *in vivo* have been described as in the previous study (Campbell *et al.*, 2012; Grisez *et al.*, 2018; Kobayashi *et al.*, 2011; Li *et al.*, 2017b). For the bone metastasis study, BALB/c‐nu mice (5–6 weeks old, 18–20 g) were anesthetized and inoculated into the left cardiac ventricle with 1 × 10^5^ luciferase‐labeled PC‐3 cells in 100 μL PBS. Bone metastases were monitored by bioluminescent imaging (BLI). Osteolytic lesions were identified on radiographs as radiolucent lesions in the bone. Each bone metastasis was scored based on the following criteria: 0, no metastasis; 1, bone lesion covering < 1/4 of the bone width; 2, bone lesion involving 1/4 ~ 1/2 of the bone width; 3, bone lesion across 1/2 ~ 3/4 of the bone width; and 4, bone lesion > 3/4 of the bone width. The bone metastasis score for each mouse was the sum of the scores of all bone lesions from the hind limbs. For survival studies, mice were monitored daily for signs of discomfort and were either euthanized all at one time or individually when presenting signs of distress, such as a 10% loss of body weight, paralysis, or head tilting.

### 2.11 Gene Set enrichment analysis (GSEA)

Gene set enrichment analysis (GSEA) was used in this study to determine whether a defined set of genes show significant, concordant differences between two sample groups divided by the median of PCAT7 expression in TCGA, PCAT7‐High (PCAT7‐H), and PCAT7‐Low (PCAT7‐L) groups. GSEA was performed using GSEA 2.2.1 (http://www.broadinstitute.org/gsea), and gene set was obtained from the Molecular Signatures Database v5.2 (http://software.broadinstitute.org/gsea/msigdb). The C2 and C5 gene sets’ collection was used in this study. mRNA expression profiles from 498 PCa patients in TCGA were used as input. False discovery rate (FDR) q values were calculated using 1000 permutations, and a gene set was considered significantly enriched if its normalized enrichment score (NES) has an FDR q below 0.25.

### 2.12 The Cancer Genome Atlas (TCGA)

lncRNA expression profiles of all 498 PCa tissues and 52 adjacent normal tissues were downloaded from TCGA (https://gdc.cancer.gov/). lncRNA expression of 498 PCa tissues, 52 adjacent normal tissues, 10 PCa tissues with bone metastasis (PCa/BM), and 12 PCa tissues without bone metastasis (PCa/nBM) was analyzed in this study. Patients with available information about survival status were used for survival analysis. All data analysis was performed using graphpad 7.0 (USA), Microsoft Excel (USA), and SangerBox (China, http://sangerbox.com/). All data were normalized as TPM format. Gene expression was presented as the mean value of multiple probes for each gene after log2 transformation. The lncRNA expression levels with more than or equal to twofold in PCa tissues and primary PCa tissues with bone metastasis (PCa/BM) compared with those in adjacent normal tissues and primary PCa tissues without bone metastasis (PCa/nBM), respectively, were selected for further study. *P* < 0.05 was considered statistically significant.

### 2.13 Statistical analysis

All data analyses were performed by graphpad 7.0 software. All values are reported as mean ± standard deviation (SD). Student’s *t*‐test was used to determine statistical differences between the two groups. A *P* value of < 0.05 was considered to indicate statistical significance.

## 3 Results

### 3.1 Identification of PCAT7 as a probone metastasis‐relevant lncRNA in PCa

**Figure 1:**
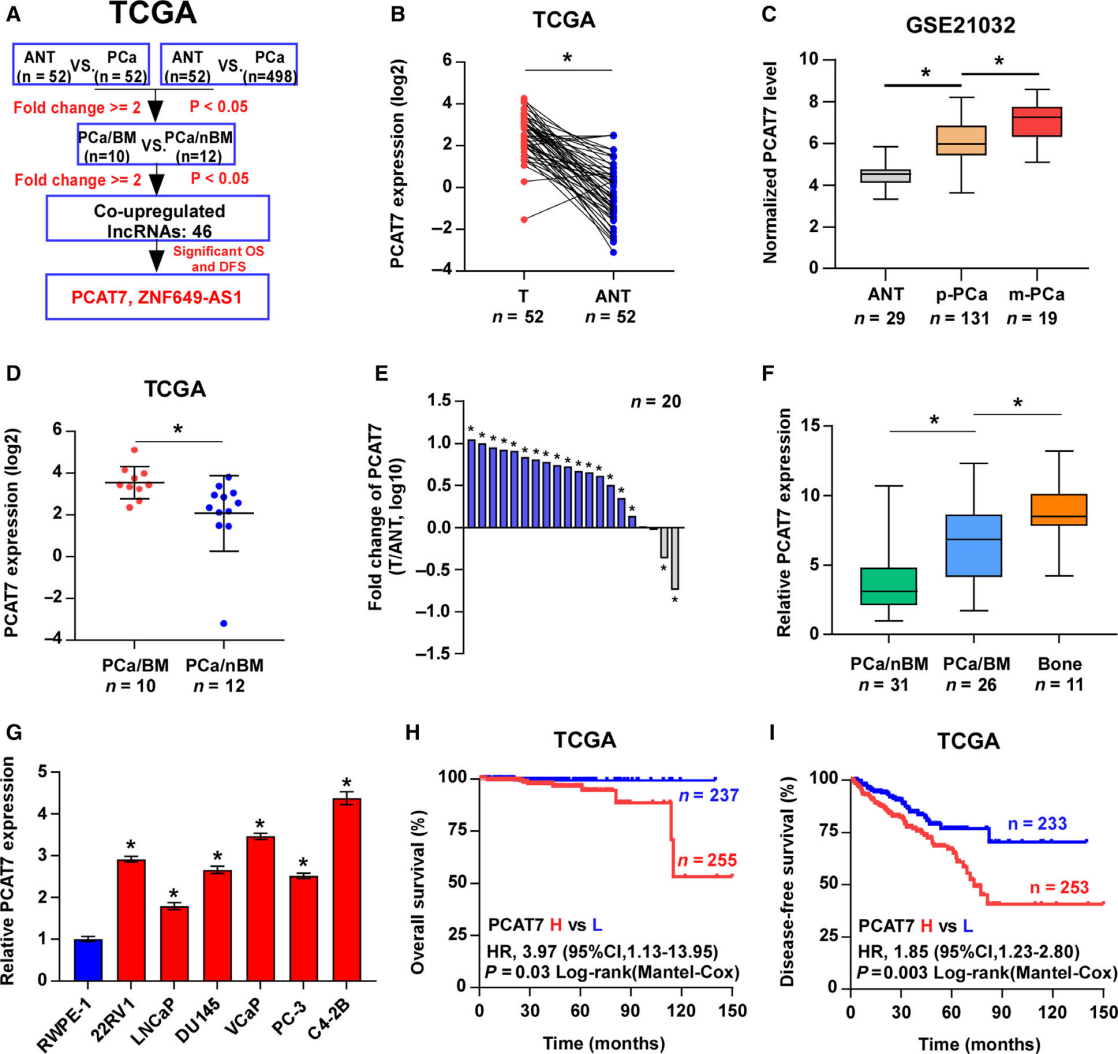
Identification of PCAT7 as a probone metastasis‐relevant lncRNA in PCa. (A) Schematic representation of PCAT7 upregulation in PCa tissues and bone metastatic PCa tissues. The screened lncRNAs were upregulated by more than twofold in PCa tissues (PCa) and PCa tissues with bone metastasis (PCa/BM) compared with adjacent normal tissues (ANT) and PCa tissues without bone metastasis (PCa/nBM), respectively, and predicted poor overall survival (OS) and disease‐free survival (DFS). (B) PCAT7 expression in 52 paired PCa tissues and their matched ANT in TCGA dataset. Data are shown as mean ± SD. *P < 0.05. (C) PCAT7 expression in ANT (*n* = 29), primary PCa tissues (p‐PCa, *n* = 131), and metastatic PCa tissues (m‐PCa, *n* = 19) in GEO dataset (http://www.ncbi.nlm.nih.gov/geo/query/acc.cgi?acc=GSE21032). Data are shown as mean ± SD. *P < 0.05. (D) PCAT7 expression in PCa/nBM (*n* = 12) and PCa/BM (*n* = 10) in TCGA dataset. Data are shown as mean ± SD. *P < 0.05. (E) Real‐time PCR analysis of the fold change of PCAT7 expression in 20 paired PCa tissues and their matched ANT. Data are shown as mean ± SD. (F) Real‐time PCR analysis of PCAT7 expression in PCa/nBM (*n* = 31), PCa/BM (*n* = 26), and metastatic bone tumors (bone tumors formed by the metastatic prostate cancer cells in bone, Bone, *n* = 11). Data are shown as mean ± SD. *P < 0.05. (G) Real‐time PCR analysis of PCAT7 expression levels in different PCa cell lines. Data are shown as mean ± SD. *P < 0.05. (H, I) Kaplan–Meier analysis of overall survival (H) and disease‐free survival (I) curves of the PCa patients stratified by PCAT7 expression in TCGA dataset.

To screen for potential probone metastasis‐related lncRNAs in PCa, the lncRNA expression profiles from The Cancer Genome Atlas (TCGA) dataset were first analyzed, and the screening rationale is depicted in Fig. 1A. Interestingly, the lncRNA, prostate cancer‐associated transcript 7 (PCAT7), was screened out to be a significant bone metastasis‐related lncRNA in PCa and was selected for further study by virtue of its nomenclature indicating its potentially critical role in PCa. The result of this inference was further supported by the TCGA and GEO (http://www.ncbi.nlm.nih.gov/geo/query/acc.cgi?acc=GSE21032) datasets that found PCAT7 to be markedly elevated in PCa tissues compared with adjacent normal tissues (ANT) (Fig. 1B,C and Fig. S1a). More importantly, the overexpression of PCAT7 was observed in PCa tissues derived from metastatic sites (Fig. 1C), including bone (Fig. 1D) via analyzing PCa datasets from TCGA and http://www.ncbi.nlm.nih.gov/geo/query/acc.cgi?acc=GSE21032. PCAT7 expression was further validated in our sample of 20 paired fresh tissues of PCa, as well as in the 31 PCa tissues without bone metastasis (PCa/nBM), 26 PCa tissues with bone metastasis (PCa/BM), and 11 metastatic bone tumor tissues (bone tumors formed by the metastatic prostate cancer cells in bone, Bone). Consistently, the results indicated that PCAT7 was elevated in PCa tissues relative to that in ANT (Fig. 1E), and gradually increased from PCa/nBM and PCa/BM to metastatic bone tumor tissues (Fig. 1F). The expression of PCAT7 in cell lines of PCa was also examined. Consistent with its expression pattern in clinical PCa tissues, PCAT7 expression was found to be differentially upregulated in PCa cells relative to that in RWPE‐1 cells (normal prostate cell; Fig. 1G). Subsequent analyses revealed that the upregulation of PCAT7 was positively associated with advanced pathological characteristics, including Gleason score, tumor volume, lymph node metastasis, and bone metastasis status (Table S4 and Fig. S1b–e), and showed poor overall and disease‐free survival of PCa patients (Fig. 1H,I). Hence, above findings indicate that the upregulation of PCAT7 could be involved in bone metastasis of PCa.

### 3.2 PCAT7 promotes PCa bone metastasis *in vivo*

**Figure 2:**
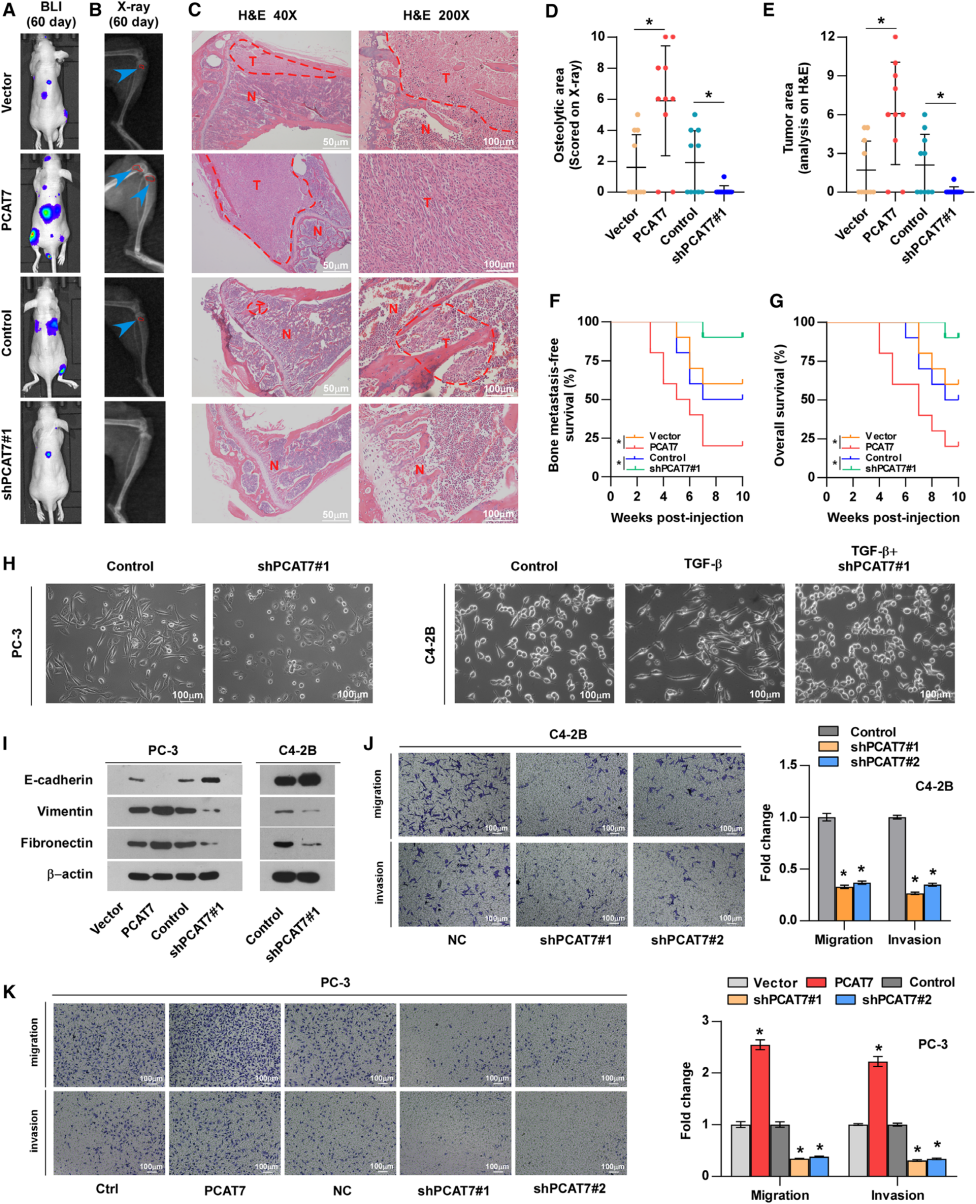
PCAT7 promotes bone metastasis of PCa cells. (A) Representative BLI signal of bone metastasis of a mouse from the indicated groups of mice on day 60 (*n* = 10 per group). (B) Representative radiographic images of bone metastases in the mice groups injected with vector, PCAT7‐overexpressing, control, and PCAT7‐knockdown PC‐3 cells, respectively (arrows indicate osteolytic lesions). (C) Representative H&E‐stained sections of tibias from the indicated mouse (T, tumor; N, the adjacent nontumor tissues). (D) The sum of bone metastasis scores for each mouse in the indicated mice groups. Data are shown as mean ± SD. *P < 0.05. (E) Histomorphometric analysis of tumor areas in the hind limbs of the indicated mice groups. Data are shown as mean ± SD. *P < 0.05. (F, G) Kaplan–Meier analysis of mouse bone metastasis‐free (F) and overall (G) survival in the indicated mice groups. (H) Silencing PCAT7 converted a stick‐like or long spindle‐shaped mesenchymal profile to a cobblestone‐like or a short spindle‐shaped epithelial morphology in PC‐3 cells (left panel). Silencing PCAT7 converted a stick‐like or long spindle‐shaped mesenchymal profile to a cobblestone‐like or a short spindle‐shaped epithelial morphology in C4‐2B cells treated with TGF‐β (right panel, 5 ng·mL^−1^ for 72 h). (I) Western blot analysis of E‐cadherin, vimentin, and fibronectin expression in the indicated PCa cells. (J, K) Upregulating PCAT7 increased, while silencing PCAT7 inhibited invasion and migration abilities in PCa cells. Data are shown as mean ± SD. *P < 0.05.

To determine whether PCAT7 serves as a bone metastasis‐related lncRNA in PCa, an *in vivo* bone metastasis model was employed, in which the PCAT7‐overexpressing, PCAT7‐downregulated, and corresponding control PC‐3 cells labeled with luciferase were directly inoculated into the left cardiac ventricle of nude mice (Fig. S2a). As shown in Fig. 2A–C and Fig. S2b, upregulation of PCAT7 promoted, while silencing PCAT7 repressed the bone metastasis ability of PCa cells, as determined using bioluminescence imaging (BLI), H&E staining, and X‐ray. Moreover, upregulation of PCAT7 increased bone metastatic loci (X‐ray) and tumor burden (H&E), and shortened bone metastasis and overall survival of the mice (Fig. 2D–G), while silencing PCAT7 showed an opposite effect (Fig. 2D–G). Notably, PCAT7 overexpression was found to accelerate, while PCAT7 knockdown delayed the onset of bone metastasis in mice (Fig. 2F). Collectively, these results suggest that PCAT7 promotes PCa bone metastasis *in vivo.*

### 3.3 PCAT7 promotes EMT, migration, and invasion *in vitro*

In order to further study the specific functions of PCAT7 in bone metastasis of PCa, the results of gene set enrichment analysis (GSEA) based on TCGA dataset showed significant enrichment of tumor metastasis and EMT‐related gene modules in sample group with high expression of PCAT7 (PCAT7‐H group), which indicated that PCAT7 may promote EMT and tumor metastasis (Fig. S2c). Subsequent experiments revealed that PCAT7 knockdown inhibited EMT in PC‐3 cells (Fig. 2H). Meanwhile, the results indicated that TGF‐β treatment reinforced the mesenchymal phenotype of PC‐3 cells (Fig. S2d). Moreover, we further silenced PCAT7 in TGF‐β‐treated PC‐3 cells and found that silencing PCAT7 partially rescued the mesenchymal phenotype induced by TGF‐β in PC‐3 cells (Fig. S2d). Since C4‐2B cells are predominated of an epithelial cell phenotype, TGF‐β was utilized to induce a mesenchymal phenotype for C4‐2B cells (Fig. 2H). Here, in C4‐2B cells, we observed that TGF‐β dramatically upregulated PCAT7 expression (Fig. S2e). More importantly, we further silenced PCAT7 in C4‐2B cells with the treatment of TGF‐β and observed that the mesenchymal phenotype of C4‐2B cells induced by TGF‐β was reversed by the knockdown of PCAT7 (Fig. 2H). These findings suggest that PCAT7 may serve as a downstream effector of TGF‐β signaling, at least in TGF‐β‐induced EMT in C4‐2B cells. The results of western blotting indicated that the overexpression of PCAT7 reduced the expression of E‐cadherin (epithelial marker), but upregulated the expression of vimentin and fibronectin (mesenchymal markers) in PC‐3 cells (Fig. 2I); conversely, PCAT7 knockdown enhanced E‐cadherin expression, but decreased vimentin and fibronectin expression in PCa cells (Fig. 2I). As shown in Fig. 2J,K, PCAT7 overexpression significantly promoted PCa cell migration and invasion, whereas PCAT7 knockdown had an opposite effect. Our findings suggest that PCAT7 promotes EMT, migration, and invasion of PCa cells *in vitro*.

### 3.4 PCAT7 sponges miR‐324‐5p in PCa

**Figure 3:**
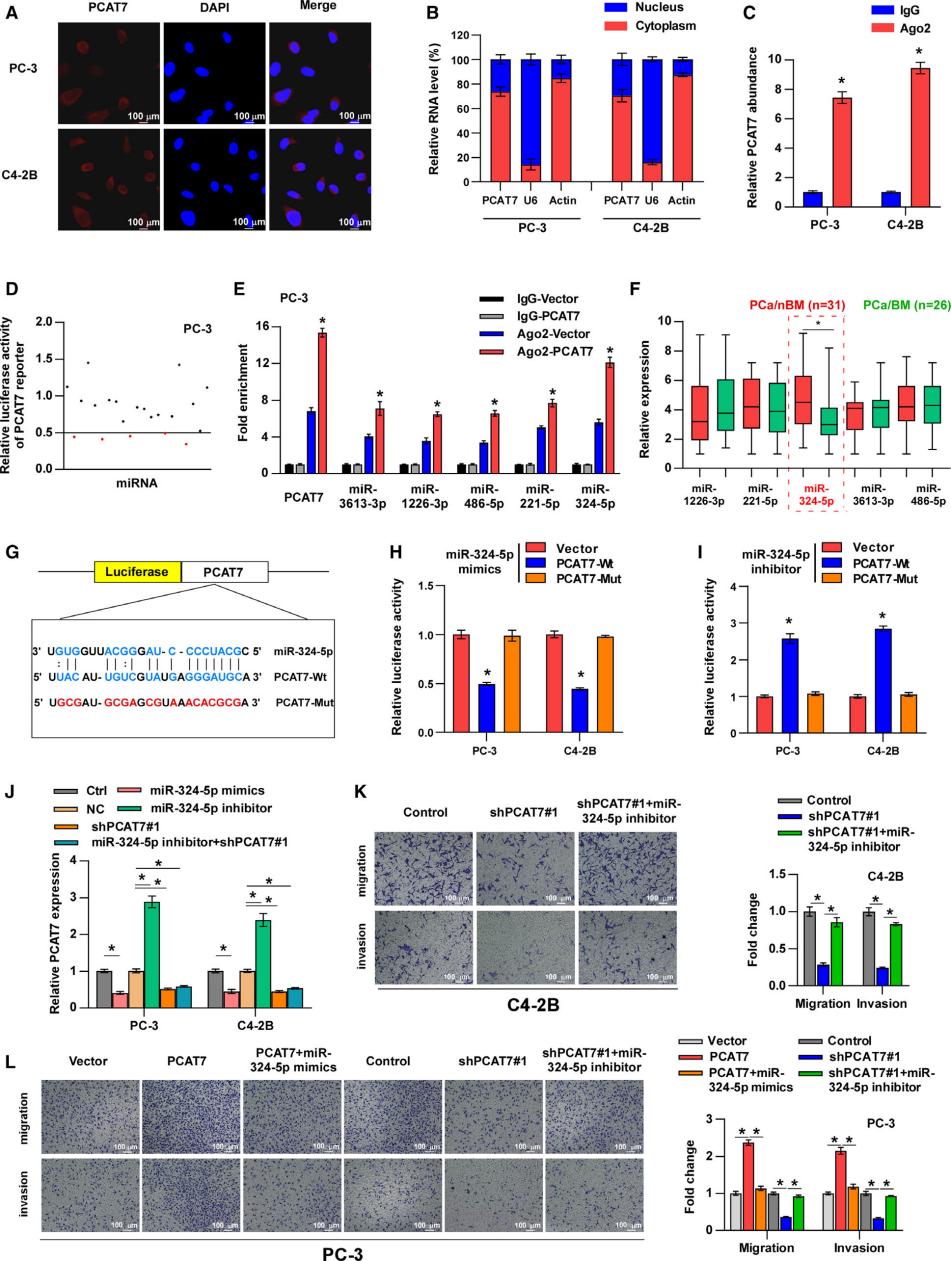
PCAT7 acts as a competitive endogenous RNA for miR‐324‐5p in PCa. (A) RNA FISH showed that PCAT7 was mainly located in cytoplasm of PCa cells. (B) Nuclear–cytoplasmic fractionation assays revealed that PCAT7 was abundant in cytoplasm of PCa cells. U6 and actin were used as positive controls in nucleus and cytoplasm, respectively. (C) Ago2 RNA immunoprecipitation (RIP) assay for the amount of PCAT7 in PC‐3 cell. Data are shown as mean ± SD. *P < 0.05. (D) Luciferase reporter assay for the luciferase activity of PCAT7 reporter in PC‐3 cells transfected with a library of 23 miRNA mimics to identify miRNAs that were able to bind to the PCAT7 sequence. miRNAs that inhibited luciferase activity by at least 50% are indicated by red dots. (E) Ago2 RNA immunoprecipitation (RIP) assay for the amount of PCAT7, miR‐3613‐3p, miR‐1226‐3p, miR‐485‐5p, miR‐221‐5p, and miR‐324‐5p in PC‐3 vector and PC‐3‐PCAT7 cells. Data are shown as mean ± SD. *P < 0.05. (F) The expression of five miRNAs in PCa/nBM (*n* = 31) and PCa/BM (*n* = 26) in our clinical samples. Data are shown as mean ± SD. *P < 0.05. (G) The predicted miR‐324‐5p binding sites in PCAT7 (PCAT7‐Wt) and the designed mutant sequence (PCAT7‐Mut) were indicated. (H) Luciferase reporter assay of PCa cells treated with miR‐324‐5p mimics and co‐transfected with PCAT7‐Wt or PCAT7‐Mut. Data are shown as mean ± SD. *P < 0.05. (I) Luciferase reporter assay of PCa cells treated with miR‐324‐5p inhibitor and co‐transfected with PCAT7‐Wt or PCAT7‐Mut. Data are shown as mean ± SD. *P < 0.05. (J) Real‐time PCR analysis of PCAT7 expression levels in the indicated groups. Data are shown as mean ± SD. *P < 0.05. (K, L) Transwell assay and Matrigel invasion assay to examine the effect of PCAT7 and miR‐324‐5p on cell migration and invasion of PCa cells. Data are shown as mean ± SD. *P < 0.05.

The protein‐coding potential of PCAT7 was predicted by the online tool, *phylocsf* (https://omictools.com/phylocsf-tool), and the results support the finding that PACT7 is noncoding. To reveal the specific mechanism of PCAT7 in PCa bone metastasis, we performed RNA FISH and nuclear–cytoplasmic fractionation assays to determine the subcellular localization of PCAT7. The results demonstrated that PCAT7 was mainly detected in the cytoplasm of PCa cells (Fig. 3A–B). The most common role of cytoplasmic lncRNA is to regulate mRNAs via sponging miRNAs (Zhao *et al.*, 2019), suggesting that PCAT7 may be involved in PCa bone metastasis in a ceRNA manner. In order to confirm this suggestion, the occupancy of Ago2 in the region of PCAT7 was further investigated. Ago2, the core of the RNA‐induced silencing complex (RISC), is required for miRNA‐mediated gene silencing, and potential miRNA targets can be isolated from this complex (Nie *et al.*, 2016). As shown in Fig. 3C, we found that PCAT7 was specifically enriched in the RISC via Ago2 RIP assay, implying the potential of PCAT7 to function as a ceRNA. Then, twenty‐three potential target miRNAs of PCAT7 were predicted using lncBASE and lncRNASNP2 (Fig. S3a). A miRNA mimic library was used to screen out potential miRNAs with a high affinity to PCAT7, and the results showed that five miRNAs, miR‐1226‐3p, miR‐221‐5p, miR‐324‐5p, miR‐3613‐3p, and miR‐486‐5p, decreased at least half of PCAT7 reporter’s luciferase activity in PC‐3 cells (Fig. 3D). RIP assay demonstrated that the upregulation of PCAT7 differentially enhanced, while silencing PCAT7 decreased the enrichment of the five miRNAs in the RISC (Fig. 3E and Fig. S3b,c). qRT‐PCR assays showed that PCAT7 overexpression decreased, while silencing PCAT7 increased their expressions in PCa cells (Fig. S3d). It is noteworthy that miR‐324‐5p was found to have the largest fold changes no matter what the enrichment and expression levels among these five miRNAs under the regulation of PCAT7, indicating that miR‐324‐5p may be more likely to participate in PCAT7‐induced bone metastasis of PCa. This hypothesis was further supported by the finding that miR‐324‐5p was identified as the only miRNA to be significantly downregulated in PCa/BM compared with PCa/nBM in the TCGA dataset and our clinical PCa samples (Fig. 3F and Fig. S3e). Furthermore, miR‐324‐5p was dramatically decreased in metastatic PCa tissues in the GEO dataset (Fig. S3f), further supporting the notion that low expression of miR‐324‐5p is related to the metastatic propensity of PCa. Then, that PCAT7 binds directly to miR‐324‐5p was confirmed by luciferase reporter assays. miR‐324‐5p mimics were found to inhibit the luciferase activity of PCAT7‐Wt reporter relative to the vector group, while PCAT7‐Mut reporter’s luciferase activity was not affected (Fig. 3G,H). In contrast, inhibition of miR‐324‐5p had an inverse effect (Fig. 3I). In addition, miR‐324‐5p mimics downregulated, whereas miR‐324‐5p inhibitor upregulated the expression of PCAT7 in PCa cells (Fig. 3J). Importantly, our results further revealed that miR‐324‐5p inhibitor did not significantly reverse the knockdown of PCAT7 by the shPCAT7#1, which indicated that miR‐324‐5p inhibitor could not effectively inhibit the shPCAT7#1 (Fig. 3J). The *in vitro* experiments showed that miR‐324‐5p mimics partially reversed the promotive effect caused by PCAT7 overexpression, whereas the miR‐324‐5p inhibitor attenuated the inhibitory effect of silencing PCAT7 on the migration and invasion of PCa cells (Fig. 3K,L). Collectively, above findings demonstrate that PCAT7 promotes PCa bone metastasis by acting as a ceRNA via sponging miR‐324‐5p.

### 3.5 PCAT7 promotes bone metastasis by activating TGF‐β signaling via sponging miR‐324‐5p

**Figure 4:**
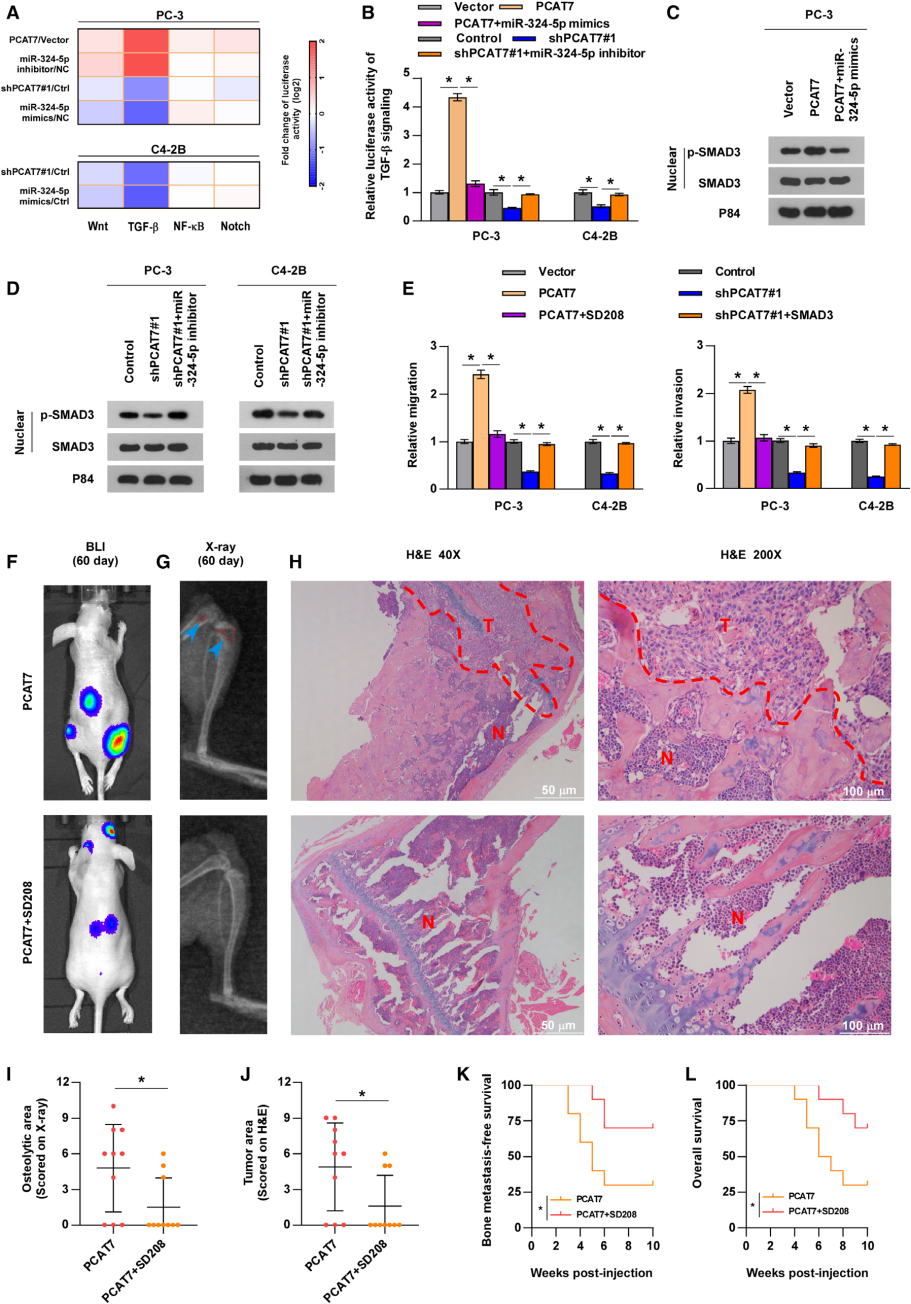
PCAT7 promotes bone metastasis by activating TGF‐β signaling via sponging miR‐324‐5p. (A) Luciferase reporter assay was performed to examine the effect of PCAT7 overexpression, PCAT7 knockdown, miR‐324‐5p mimics, and inhibitor on the luciferase activity of Wnt, TGF‐β, NF‐κB, and Notch pathway. (B) Luciferase reporter assay for the luciferase activity of TGF‐β signaling in the indicated groups. Data are shown as mean ± SD. *P < 0.05. (C, D) Western blot analysis of p‐SMAD3 and SMAD3 expression in the indicated PCa cells. (E) Transwell assay and Matrigel invasion assay to show the migration and invasion ability of PCa cells in the indicated groups. Data are shown as mean ± SD. *P < 0.05. (F) Representative BLI signal of bone metastasis of a mouse from the indicated groups of mice on day 60 (*n* = 10 per group). (G) Representative radiographic images of bone metastases in the mice groups injected with PCAT7‐overexpressing PC‐3 cells received SD208 (50 mg·kg^−1^·day^−1^) or its vehicle (arrows indicate osteolytic lesions). (H) Representative H&E‐stained sections of tibias from the indicated mouse (T, tumor; N, the adjacent nontumor tissues). (I) The sum of bone metastasis scores for each mouse in the indicated mouse groups. Data are shown as mean ± SD. *P < 0.05. (J) Histomorphometric analysis of tumor areas in the hind limbs of the indicated mice groups. Data are shown as mean ± SD. *P < 0.05. (K, L) Kaplan–Meier analysis of mouse bone metastasis‐free (K) and overall (L) survival in the indicated mice groups. *P < 0.05.

In order to determine the molecular mechanism that underlies the PCAT7/miR‐324‐5p axis in PCa bone metastasis, luciferase reporter assays were performed to assess the function of PCAT7 and miR‐324‐5p in multiple well‐known pathways that contribute to bone metastasis of PCa, including the Notch (Zayzafoon *et al.*, 2004), TGF‐β (Dai *et al.*, 2017), Wnt (Ren *et al.*, 2019), and NF‐κB (Ren *et al.*, 2017) pathways. As shown in Fig. 4A, both PCAT7 overexpression and miR‐324‐5p inhibition potently enhanced the luciferase activity of TGF‐β signaling, whereas silencing PCAT7 or miR‐324‐5p mimics strongly inhibited TGF‐β signaling’s luciferase activity. The luciferase activities of Wnt, NF‐kB, and Notch were not significantly affected by a change in the expression of PCAT7 or miR‐324‐5p (Fig. 4A). Notably, GSEA of the PCa samples in TCGA also revealed significant enrichment of the TGF‐β signaling in samples with a high expression of PCAT7 (Fig. S4a). Our findings indicate that PCAT7 activates TGF‐β signaling via sponging miR‐324‐5p. Undoubtedly, miR‐324‐5p mimics reversed the promoting effect of overexpressing PCAT7 on the luciferase activity of TGF‐β signaling, while PCAT7 downregulation showed a suppressive effect on TGF‐β signaling’s luciferase activity, which was abrogated by miR‐324‐5p inhibitor (Fig. 4B). A similar effect of miR‐324‐5p‐mediating role of PCAT7 in TGF‐β signaling was observed at the p‐SMAD3 level (Fig. 4C,D). In addition, the expression of MMP13, PTHRP, CTGF, COL1A1, and NEDD9 that are classic genes related to bone metastasis in TGF‐β pathway was upregulated by PCAT7 overexpression, whereas downregulated by PCAT7 knockdown (Fig. S4b). Thus, our results demonstrate that PCAT7 activates TGF‐β signaling via inhibiting miR‐324‐5p.

Then, the functional role of TGF‐β signaling in PCAT7‐induced bone metastasis was further investigated. As shown in Fig. 4E, SD208, an inhibitor of TGF‐β signaling (Fournier *et al.*, 2015), reversed the promigration and pro‐invasion effect of PCAT7 overexpression on PCa cells, whereas constitutively active SMAD3 mutants (Cai *et al.*, 2017) reversed the inhibitory effect of PCAT7 knockdown on the migration and invasion of PCa cells. Moreover, SD208 significantly blocked the probone metastatic function of PC‐3 cells stably overexpressing PCAT7 and prolonged overall and bone metastasis‐free survival of the mouse (Fig. 4F–L). Taken together, these findings suggest that PCAT7 activates TGF‐β signaling by sponging miR‐324‐5p to facilitate PCa bone metastasis.

### 3.6 PCAT7 disrupts miR‐324‐5p‐mediated suppression of TGFBR1

**Figure 5:**
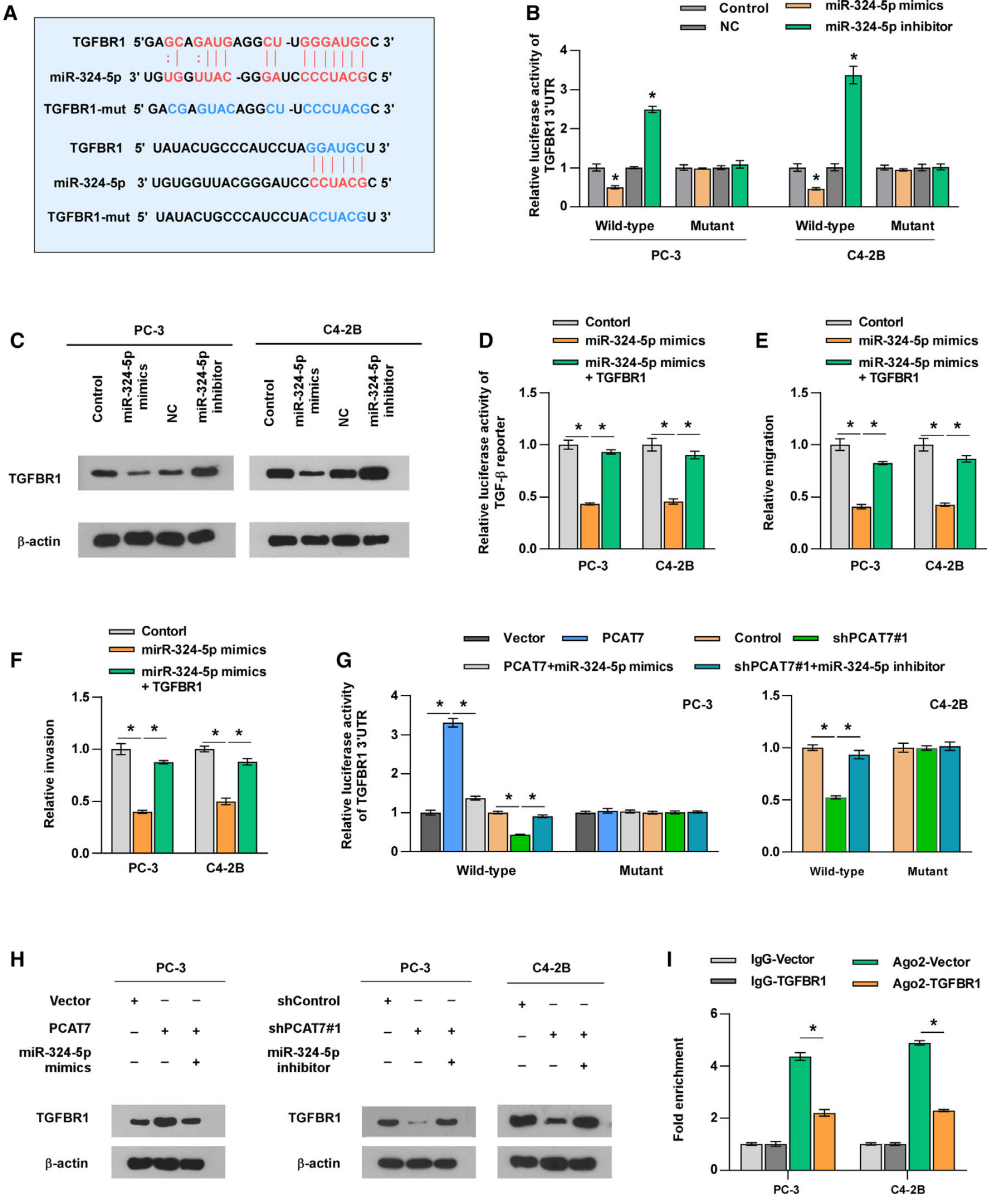
PCAT7 disrupts miR‐324‐5p‐mediated suppression on TGFBR1. (A) The predicted miR‐324‐5p binding sites in TGFBR1 (TGFBR1‐Wt) and the designed mutant sequence (TGFBR1‐Mut) were indicated. (B) Luciferase reporter assay for the luciferase activity of TGFBR1‐3′‐UTR‐Wt and TGFBR1‐3′‐UTR‐Mut reporters in the presence of miR‐324‐5p mimics or miR‐324‐5p inhibitor in PCa cells. Data are shown as mean ± SD. *P < 0.05. (C) Western blot analysis showed miR‐324‐5p mimics decreased, while miR‐324‐5p inhibitor dramatically increased TGFBR1 protein level in PCa cells. (D) Luciferase reporter assay showed the impact of miR‐324‐5p mimics and TGFBR1 on the luciferase activity of TGF‐β signaling. Data are shown as mean ± SD. *P < 0.05. (E, F) Transwell assay (E) and Matrigel invasion assay (F) to show the migration and invasion ability of PCa cells in the indicated groups. Data are shown as mean ± SD. *P < 0.05. (G) Luciferase reporter assay of TGFBR1‐3′‐UTR‐Wt and TGFBR1‐3′‐UTR‐Mut reporters in PCa cells with the expression of PCAT7 and miR‐324‐5p changed. Data are shown as mean ± SD. *P < 0.05. (H) Western blot analysis for TGFBR1 in PCa cells with the expression of PCAT7 and miR‐324‐5p changed. (I) Ago2 RNA immunoprecipitation (RIP) assay for the amount of PCAT7 in the indicated group. Data are shown as mean ± SD. *P < 0.05.

Through analyzing Starbase and miDIP, the results implied that TGFBR1 may be the target of miR‐324‐5p (Fig. 5A). Subsequently, luciferase reporter assays revealed that miR‐324‐5p mimics reduced, whereas miR‐324‐5p inhibitor enhanced the luciferase activity of wild‐type 3′‐UTR of TGFBR1. However, the luciferase activity of mutant 3′‐UTR of TGFBR1 was not affected (Fig. 5B). qRT‐PCR and western blotting assays revealed that miR‐324‐5p mimics inhibited, whereas miR‐324‐5p inhibitor enhanced the mRNA and protein expression of TGFBR1 (Fig. 5C and Fig. S5a). It is noteworthy that TGFBR1 was upregulated in PCa/BM relative to that in PCa/nBM in TCGA, which was further confirmed by the results obtained from our samples (Fig. S5b). Our results further showed that TGFBR1 overexpression reversed the suppressive effect of miR‐324‐5p mimics on TGF‐β signaling (Fig. 5D) and the migration and invasion of PCa cells (Fig. 5E,F). Therefore, the abovementioned findings demonstrate that miR‐324‐5p targets TGFBR1 in PCa cells.

Since PCAT7 acts as a sponge for miR‐324‐5p, it is conceivable that PCAT7 should upregulate TGFBR1 expression. As expected, the overexpression of PCAT7 increased, while silencing PCAT7 reduced the luciferase activity of wild‐type TGFBR1‐3′‐UTR and the protein expression of TGFBR1 (Fig. 5G,H). Moreover, miR‐324‐5p mimics reversed the increase in the luciferase activity of wild‐type TGFBR1‐3′‐UTR and TGFBR1 expression caused by PCAT7 overexpression (Fig. 5G,H). Conversely, miR‐324‐5p inhibitor reversed the suppressive effect of PCAT7 downregulation on wild‐type TGFBR1‐3′‐UTR and TGFBR1 expression (Fig. 5G,H). Meanwhile, the mutant TGFBR1‐3′‐UTR’s luciferase activity was not affected by changed expression of PCAT7 and miR‐324‐5p (Fig. 5G). More importantly, upregulation of TGFBR1 was found to reduce enrichment of PCAT7 in Ago2 (Fig. 5I). These results indicate that PCAT7 upregulates TGFBR1 by disrupting the suppressive effect of miR‐324‐5p on TGFBR1 expression via competitively binding to miR‐324‐5p, which further activates TGF‐β signaling and promotes PCa bone metastasis.

### 3.7 SP1 promotes PCAT7 transcription in PCa cells

**Figure 6:**
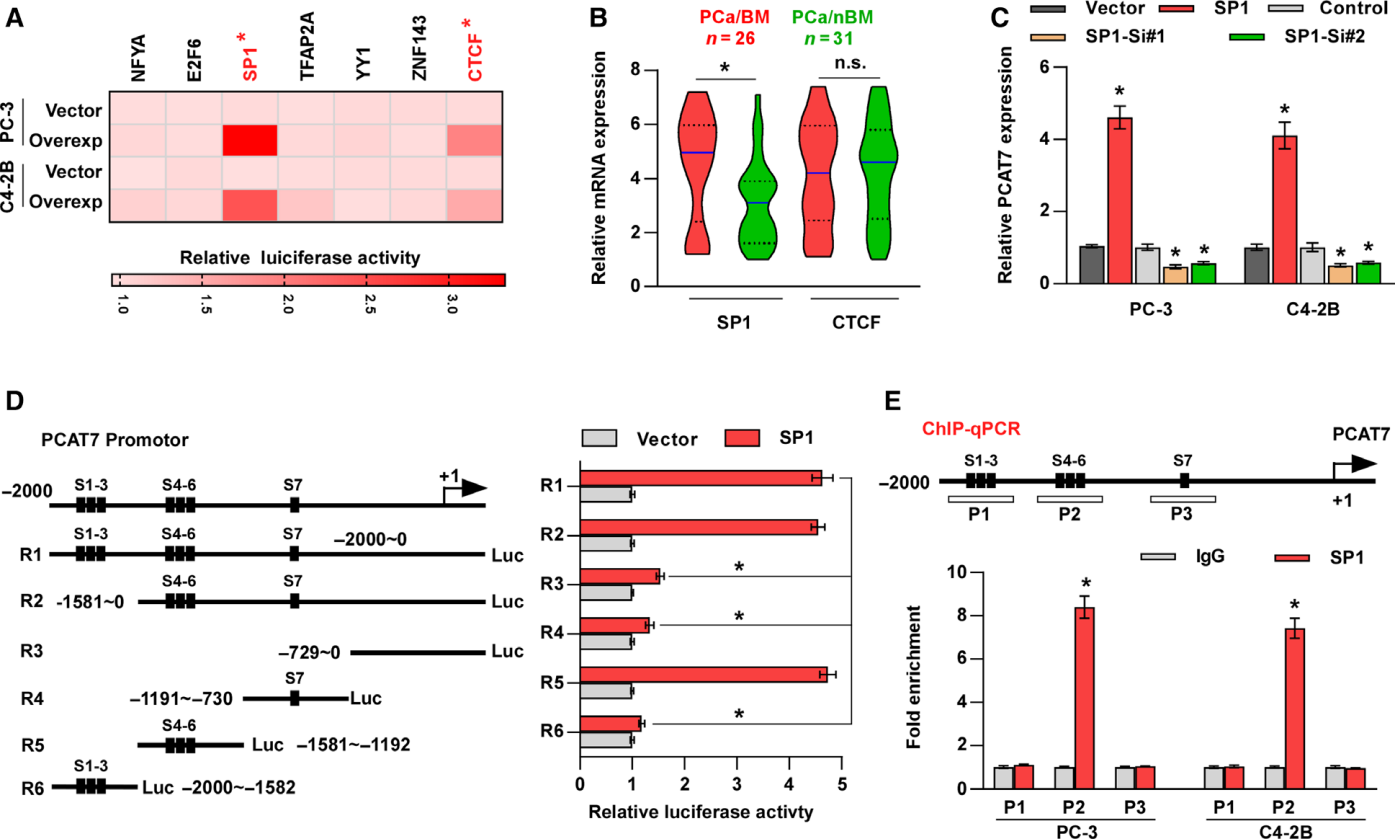
SP1 promotes PCAT7 transcription in PCa cells. (A) Luciferase reporter assay for the luciferase activity of PCAT7 reporter regulated by seven transcriptional factors. Overexp: overexpression. (B) SP1 and CTCF expression in PCa/nBM (*n* = 31) and PCa/BM (*n* = 26) in our clinical samples. Data are shown as mean ± SD. *P < 0.05. (C) PCAT7 expression in the indicated PCa cells. Data are shown as mean ± SD. *P < 0.05. (D) Schematic representation of constructs for PCAT7 promotor (left panel). Luciferase activities of 6 truncated constructs in PC‐3 vector and PC‐3 SP1 cells (right panel). Data are shown as mean ± SD. *P < 0.05. (E) Prediction and validation of possible SP1‐binding sites. Data are shown as mean ± SD. *P < 0.05.

As demonstrated above, PCAT7 upregulates TGFBR1 via sponging miR‐324‐5p, which further activates TGF‐β signaling. Intriguingly, our results showed that TGF‐β enhanced PCAT7 expression in PCa cells (Figs S2b and S6a), indicating the existence of a positive feed‐forward loop between PCAT7 and TGF‐β signaling. As is well known, TGF‐β signaling regulates downstream gene expression via formation of complexes in the nucleus between SMADs and DNA‐binding cofactors, such as SP1, or with transcriptional coactivators or corepressors (Shi and Massague, 2003). Therefore, in order to explore the underlying mechanism of TGF‐β‐induced upregulation of PCAT7, we further used JASPAR combined with the results of ChIP‐seq from ENCODE to predict transcription factors that are involved in transcriptional regulation‐mediated PCAT7 upregulation and found seven potential transcription factor candidates. Among these, we found that the overexpression of SP1, an important DNA‐binding cofactor of the SMAD complex, potently enhanced, while CTCF slightly enhanced the luciferase activity of the PCAT7 promoter (Fig. 6A). Importantly, SP1, but not CTCF, expression was dramatically upregulated in PCa/BM relative to that in PCa/nBM in our samples and TCGA dataset (Fig. 6B and Fig. S6b). Meanwhile, SP1 expression showed a positive correlation with PCAT7 expression in our PCa samples and TCGA dataset (Fig. S6c). Therefore, SP1 was selected for further investigation. qRT‐PCR assays revealed that SP1 overexpression upregulated, whereas SP1 knockdown reduced PCAT7 expression in PCa cells (Fig. 6C). Subsequently, we found 7 putative sites in the promoter region of PCAT7 which may specifically bind to SP1 (Fig. 6D). To confirm that PCAT7 was a transcriptional target of SP1, we conducted several truncations of the PCAT7 promoter and measured their luciferase activities (Fig. 6D). The results indicated that the fragment (−1581 ~ −1192 nt), containing sites 4–6, was essential for the transcription of PCAT7 mediated by SP1 (Fig. 6D). In order to identify the specific site that binds to SP1, we established four mutants for sites 4–6 and found that the mutation on sites 5 and 6 decreased the luciferase activity of the PCAT7 promoter, which implied that sites 5 and 6 are responsible for the transcriptional activation of PCAT7 regulated by SP1 (Fig. S6d). Furthermore, the enrichment of SP1 on the P2 fragment of the PCAT7 promoter containing sites 5–6 was confirmed through ChIP assay (Fig. 6E). Collectively, above findings demonstrate that SP1 transcriptionally upregulates PCAT7 expression in PCa.

### 3.8 SP1 mediates the positive feedback loop between PCAT7 and TGF‐β signaling

**Figure 7:**
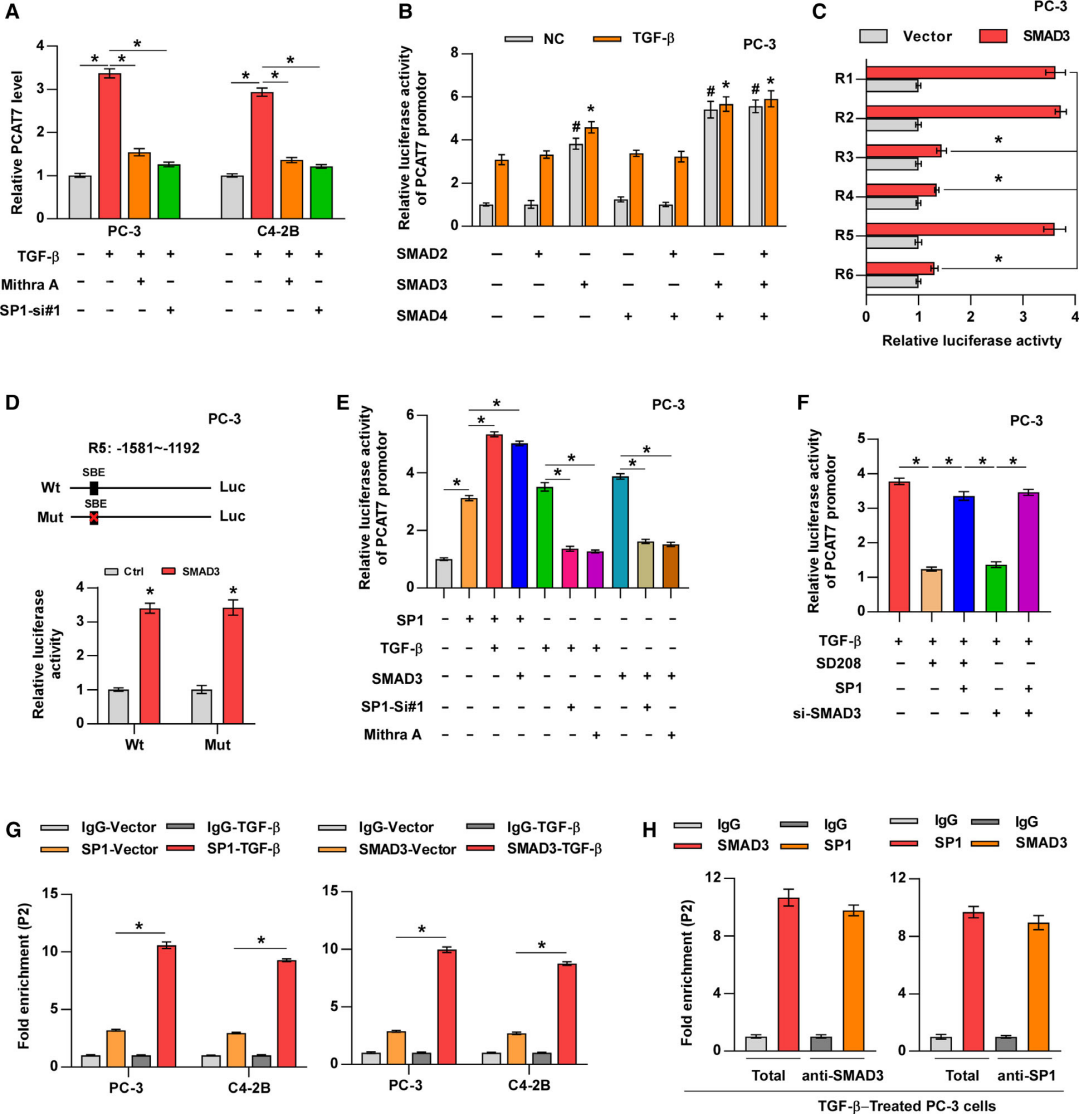
SP1 mediates a positive feedback loop between PCAT7 and TGF‐β signaling. (A) PCAT7 expression in the indicated groups. Data are shown as mean ± SD. *P < 0.05. (B) Luciferase activity of the PCAT7 promotor in PC‐3 cells transfected with SMAD2, SMAD3, or SMAD4 and treated ± TGF‐β. Data are shown as mean ± SD. ^#^,*P < 0.05. (C) Luciferase activities of six truncated constructs in PC‐3 vector and PC‐3 SMAD3 cells. Data are shown as mean ± SD. *P < 0.05. (D) Luciferase activities of PCAT7‐Wt or PCAT7‐Mut promotor in PC‐3 vector and PC‐3 SMAD3 cells. Data are shown as mean ± SD. *P < 0.05. (E, F) Luciferase activities of PCAT7 promotor in the indicated groups. Data are shown as mean ± SD. *P < 0.05. (G) Chromatin immunoprecipitation assay showed the binding capacity of SP1 and SMAD3 to the PCAT7 promoter (P2) was notably higher in the TGF‐β‐treated group than in the control group. Data are shown as mean ± SD. *P < 0.05. (H) The co‐occupancy of SP1 and SMAD3 on PCAT7 promoter was identified via ChIP‐re‐ChIP assay.

Many studies have reported that TGF‐β‐dependent SMAD proteins and ubiquitous transcriptional factor SP1 can form a transcriptional complex to individually or concomitantly modulate the expression of downstream target genes (Feng *et al.*, 2000; Poncelet and Schnaper, 2001; Shi and Massague, 2003). As mentioned above and shown in Fig. 7A, ectopic TGF‐β enhanced PCAT7 expression, indicating the existence of a feed‐forward loop between PCAT7 and TGF‐β signaling. More importantly, this suppressive effect of TGF‐β on PCAT7 expression was eliminated by SP1 knockdown or mithramycin A, which inhibits the DNA‐binding ability of SP1 (Ray *et al.*, 1989), indicating that SP1 may be involved in TGF‐β signaling‐induced PCAT7 upregulation, which requires the DNA‐binding ability of SP1. Then, to determine whether SMAD proteins mediated the TGF‐β‐induced transcriptional activation of PCAT7, the effect of SMAD2, SMAD3, and SMAD4 on the promoter activity of PCAT7 was further assessed in PC‐3 cells. We observed that SMAD2 and SMAD4 both individually or in combination had no effect on PCAT7 promoter’s luciferase activity, regardless of the TGF‐β treatment status (Fig. 7B). However, SMAD3 overexpression significantly increased the basal or TGF‐β‐induced PCAT7 promoter activity, while luciferase activity reached a maximum when SMAD3 and SMAD4 were both overexpressed (Fig. 7B). Our results implied that SMAD3 may be implicated in the TGF‐β‐induced transcriptional activation of PCAT7. Then, we aimed to identify the fragment of PCAT7 promoter that is necessary for SMAD3‐mediated transcription of PCAT7. We found that SMAD3 overexpression increased the luciferase activity of PCAT7 via the R5 fragment (−1581 ~ −1192 nt), which was consistent with the regulatory region of SP1 within the promoter of PCAT7 (Fig. 7C). We further analyzed the sequence of the R5 fragment and found only one SMAD3/4 binding element (SBE), 5′‐GACA‐3′ (Shi and Massague, 2003; Fig. 7D). Surprisingly, mutation analysis revealed that SBE was not essential for PCAT7 transcription by SMAD3 (Fig. 7D), which implied that TGF‐β‐induced transcription of PCAT7 did not require the DNA‐binding ability of SMAD3. However, the luciferase reporter assay further revealed that both ectopic TGF‐β and the overexpression of SMAD3 reinforced the transcriptional upregulation of SP1 in PCAT7, and the stimulatory effects of TGF‐β and the overexpression of SMAD3 on the luciferase activity of the PCAT7 promoter were dramatically blocked by SP1 knockdown or mithramycin A (Fig. 7E). Conversely, the overexpression of SP1 remarkably abrogated the suppressive effect of SD208 or silencing SMAD3 on PCAT7 promoter’s luciferase activity in TGF‐β‐treated PC‐3 cells (Fig. 7F). Thus, these findings imply that SP1 transcriptionally upregulates PCAT7 independently of TGF‐β/SMAD3 signaling, although TGF‐β/SMAD3 pathway promotes the transcriptional regulatory efficiency of SP1 on PCAT7. The ChIP assays showed that the enrichment of SP1 and SMAD3 on P2 of the PCAT7 promotor was upregulated by the activation of TGF‐β signaling (Fig. 7G), which indicated that the TGF‐β‐induced SMAD3/SP1 complex increased the DNA‐binding ability of SP1 with the PCAT7 promotor. Subsequent ChIP‐re‐ChIP assays further demonstrated the co‐enrichment of SMAD3 and SP1 on the P2 fragment of the PCAT7 promoter containing sites 5–6, which is essential for the transcription of PCAT7 by SP1 (Fig. 7H). Collectively, these results indicate that the SMAD3/SP1 complex mediates a positive feedback loop between PCAT7 and TGF‐β signaling.

### 3.9 Clinical relevance of PCAT7‐miR‐324‐5p‐TGF‐β signaling in PCa

**Figure 8:**
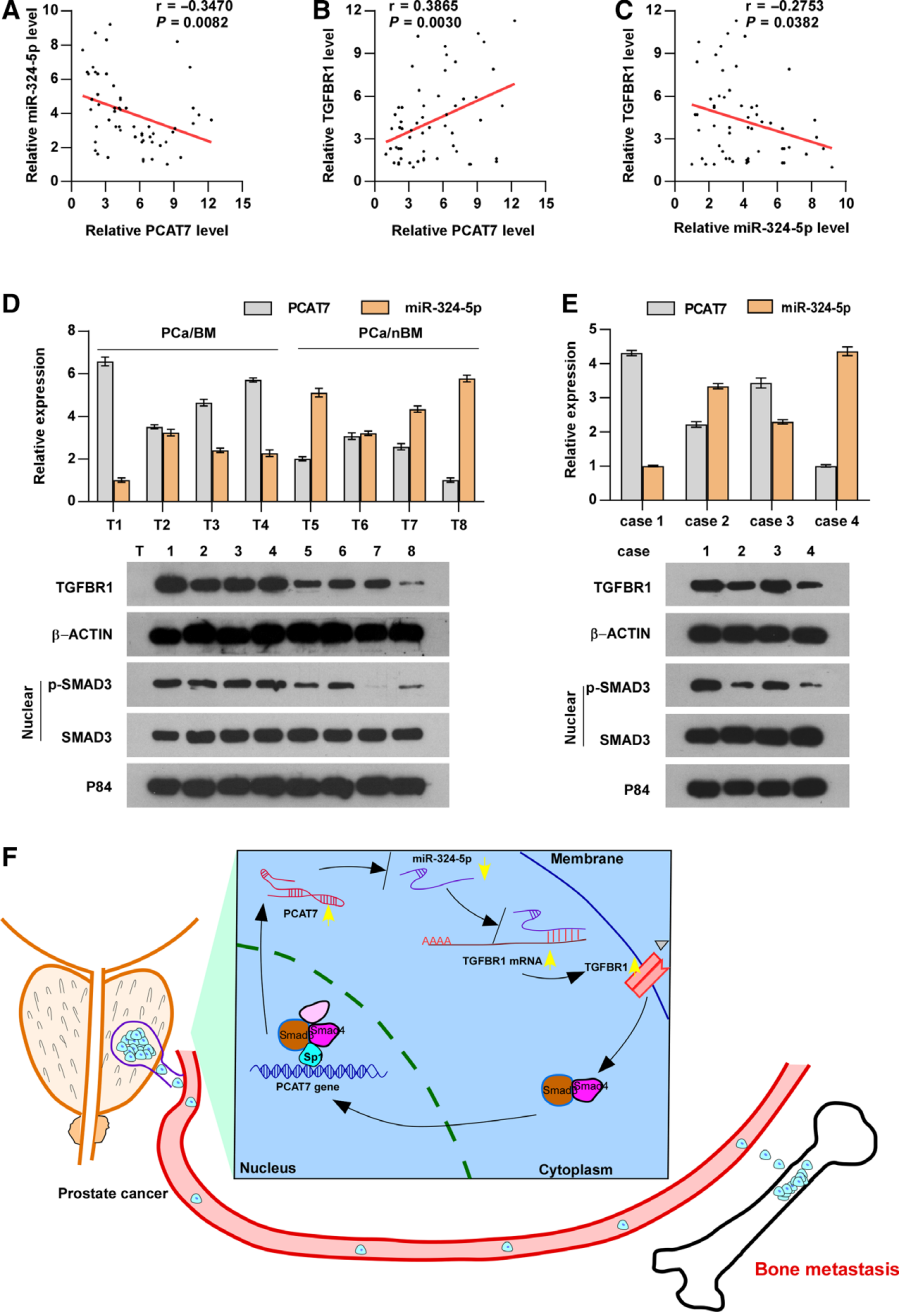
Clinical relevance of PCAT7‐miR‐324‐5p‐TGF‐β signaling in PCa. (A) PCAT7 expression was negatively correlated with miR‐324‐5p expression in PCa patients. (B) PCAT7 expression was positively correlated with TGFBR1 expression in PCa patients. (C) miR‐324‐5p expression was negatively correlated with TGFBR1 expression in PCa patients. (D) Real‐time PCR and western blot analysis of PCAT7, miR324‐5p, TGFBR1, and p‐SMAD3 expression in 4 random PCa/BM (T1‐T4) and 4 random PCa/nBM (T5‐T8). (E) Real‐time PCR and western blot analysis of PCAT7, miR324‐5p, TGFBR1, and p‐SMAD3 expression in primary PCa cells. (F) Model: SMAD3/SP1 complex‐mediated constitutive active loop between lncRNA PCAT7 and TGF‐β signaling promotes prostate cancer bone metastasis.

Given the significance of the PCAT7‐miR‐324‐5p‐TGF‐β signaling axis in bone metastasis of PCa cells, we analyzed the clinical relevance of this loop in PCa patients. We found that the expression of PCAT7 was reversely associated with the expression of miR‐324‐5p, while it was positively associated with TGFBR1 expression in PCa patients of our cohort and the TCGA cohort (Fig. 8A,B and Fig. S7a,b). The expression level of miR‐324‐5p was found to be negatively correlated with TGFBR1 mRNA levels in our PCa patients and the TCGA dataset (Fig. 8C and Fig. S7c). Furthermore, the expression of PCAT7, miR‐324‐5p, TGFBR1, p‐SMAD3, and SMAD3 was further determined in 4 random PCa/BM (T1‐T4) and 4 random PCa/nBM (T5‐T8) through qRT‐PCR and western blotting assays. We found that PCAT7, TGFBR1, and p‐SMAD3 expression in PCa/BM (T1‐4) dramatically increased, compared with those in PCa/nBM (T5‐T8); in contrast, miR‐324‐5p expression exhibited an inverse pattern (Fig. 8D). Consistently, the positive correlation of PCAT7 with TGFBR1 and p‐SMAD3, and the negative correlation of PCAT7 with miR‐324‐5p were further clinically verified in primary PCa cells reported in our previous study (Ren *et al.*, 2019) (Fig. 8E). Meanwhile, we found that miR‐324‐5p expression was reduced in PC‐3 and C4‐2B cells; conversely, the expression of PCAT7 and TGFBR1 in PC‐3 and C4‐2B cells was upregulated compared with those in RWPE‐1 cells. These findings suggested that miR‐324‐5p expression levels were inversely correlated with PCAT7 and TGFBR1 expression in PC‐3 and C4‐2B cells (Fig. S7d,e). As shown in Fig. S7f,g, we found that the protein and mRNA expression of SP1 was differentially upregulated in PCa cell lines compared with those in RWPE‐1 cells. In addition, the results of correlation analysis showed that SP1 expression was positively correlated with PCAT7 expression, but was negatively correlated with miR‐324‐5p expression in PCa cell lines (Fig. S7h–k). Collectively, our results reveal the critical role of the SMAD3/SP1 complex‐mediated constitutive active loop between TGF‐β signaling and lncRNA PCAT7 in PCa bone metastasis (Fig. 8F).

## 4 Discussions

The present study reveals the oncogenic role of lncRNA PCAT7 in bone metastasis of PCa. Our findings indicate that PCAT7 is upregulated in bone metastasis‐positive PCa tissues. Meanwhile, the upregulation of PCAT7 is associated with advanced clinicopathologic characteristics, bone metastasis status, and poor prognosis of PCa patients. Mechanistically, PCAT7 upregulates TGFBR1 expression by sponging miR‐324‐5p, which further activates TGF‐β signaling, contributing to the onset of bone metastasis in PCa. Reciprocally, the SMAD3/SP1 transcriptional complex enhances PCAT7 transcription, forming a feedback loop between PCAT7 and TGF‐β signaling. Hence, our study has uncovered a new mechanism of PCAT7‐induced activation of TGF‐β pathway, elucidating the critical role of PCAT7 in PCa bone metastasis.

Dysregulation of a variety of lncRNAs, such as PCAT1/14/19, DNACR, TTTY15, and SChLAP1, has been extensively reported in PCa, and these dysregulations contributed significantly to the aggressiveness and progression of PCa via varying mechanisms (Hua *et al.*, 2018; Lu *et al.*, 2018; Prensner *et al.*, 2013; Shang *et al.*, 2019; White *et al.*, 2017; Xiao *et al.*, 2018). However, at present, literature about the biological function of lncRNAs in PCa bone metastasis remains scanty. Recently, Li et al. have reported that lncRNA‐MAYA mediated the crosstalk between Hippo–YAP and ROR1–HER3 pathways to promote breast cancer bone metastasis. Importantly, inhibition of lncRNA‐MAYA effectively repressed bone metastasis of breast cancer (Li *et al.*, 2017a). This finding supports the idea that targeting oncogenic lncRNAs may be a promising therapeutic strategy for bone metastasis. To identify bone metastasis‐relevant oncogenic lncRNAs, the lncRNA expression profiles of PCa dataset from TCGA were analyzed and found that PCAT7, a prostate cancer‐associated lncRNA by virtue of its nomenclature, was significantly upregulated in bone metastasis‐positive PCa tissues, further validated in our PCa samples. Functional experiments showed that upregulation of PCAT7 promoted EMT, invasiveness, and bone metastasis of PCa cells. More importantly, silencing PCAT7 dramatically inhibited PCa bone metastasis. Therefore, these results indicate the probone metastatic role of PCAT7 in PCa, suggesting that PCAT7 shows promising prospects as a potential therapeutic target against bone metastasis in PCa.

Previous studies have revealed that cytoplasmic lncRNAs generally function as ceRNAs to regulate mRNA expression via sponging versatile miRNAs, thereby playing a pivotal role in cancer metastasis (Lian *et al.*, 2018; Shan *et al.*, 2018; Sun *et al.*, 2018; Wang and Kong, 2018; Yan *et al.*, 2018). In fact, lncRNAs exert their functions in several biological processes via interaction with DNA, proteins, and RNAs, which is dependent on the subcellular localization of the lncRNAs within the cells to a large extent (Chen *et al.*, 2018). In the current study, using RNA FISH and nuclear–cytoplasmic fractionation assays, we found that PCAT7 can be mainly detected in the cytoplasm of PCa cells, suggesting that PCAT7 may function as a ceRNA in bone metastasis of PCa, which is also supported by previous findings in nasopharyngeal carcinoma and nonsmall cell lung cancer (Liu *et al.*, 2017b; Liu *et al.*, 2017a). Further investigations found that PCAT7 upregulated TGFBR1 expression by functioning as a sponge for miR‐324‐5p, which constitutively activated TGF‐β signaling and promoted PCa bone metastasis. Thus, these results reveal an oncogenic mechanism that underlies the function of PCAT7 as a ceRNA, which contributes to the sustained activation of TGF‐β signaling, further facilitating the onset of bone metastasis in PCa.

Increasing evidence has shown that lncRNAs are under extensive regulation by transcription factors, which has been documented as a main mechanism that contributes to the dysregulation of lncRNAs in many types of human cancers. For instance, NF‐κB transcriptionally upregulated LINC01410 expression in gastric cancer, which further promoted the angiogenesis and metastasis of gastric cancer (Zhang *et al.*, 2018); moreover, the STAT3‐mediated upregulation of lncRNA HOXD‐AS1 contributed to lung metastasis of hepatocellular carcinoma by preventing SOX4 degradation induced by miR‐130a‐3p (Wang *et al.*, 2017). In this study, we found that SP1 transcriptionally upregulated PCAT7 via a series of functional assays and analyses using JASPAR and ENCODE. An in‐depth investigation revealed that this transcriptional upregulation role of SP1 in PCAT7 was dramatically augmented by TGF‐β/SMAD3 pathway. Importantly, our results further clarified that SP1 transcriptionally upregulated PCAT7 independent of TGF‐β/SMAD3 pathway, although TGF‐β/SMAD3 pathway promoted the transcriptional regulatory efficiency of SP1 on PCAT7. Collectively, our findings of the current study have uncovered a novel mechanism in detail by which SP1 mediates a positive feed‐forward loop between PCAT7 and TGF‐β signaling, which is reinforced by SMAD3.

The constitutive activation of TGF‐β pathway has been widely documented to participate in bone metastasis of multiple types of cancers, such as prostate cancer and breast cancer (Dai *et al.*, 2017; Fournier *et al.*, 2015; Kang *et al.*, 2005; Korpal *et al.*, 2009; Yin *et al.*, 1999), and therapies targeting TGF‐β can effectively reduce metastasis of tumor cells to bone (Hu *et al.*, 2012; Wan *et al.*, 2012). The study from Fournier et al. has shown that SD208, a specific inhibitor of TGF‐β signaling, suppressed bone metastasis of PCa by interrupting the interaction between cancer cells and the bone microenvironment (Fournier *et al.*, 2015). It seems that targeting TGF‐β signaling is an ideal antibone metastatic target in PCa. Therefore, identification of critical functional factor restraining activity of TGF‐β signaling may promote the eradication of bone metastasis in PCa. As demonstrated above, PCAT7 competitively bound to miR‐324‐5p to prevent TGFBR1 from miR‐324‐5p‐mediated degradation, contributing to the constitutive activation of TGF‐β pathway. In turn, TGF‐β signaling upregulated PCAT7 via the SMAD3/SP1 transcriptional complex, indicating the existence of a feedback loop between PCAT7 and TGF‐β signaling. Importantly, our results further showed that silencing PCAT7 disrupted the constitutive active loop of TGF‐β signaling by restoring the suppressive function of miR‐324‐5p on TGFBR1, which dramatically repressed PCa cell bone metastasis. Hence, this study infers that PCAT7 may serve as a promising therapeutic target against bone metastasis of PCa to disrupt the constitutive active loop of TGF‐β signaling.

## 5 Conclusions

In summary, this study, for the first time, uncovers the probone metastatic function of PCAT7, which forms a positive feedback loop with TGF‐β signaling in PCa, indicating PCAT7 may serve as a promising therapeutic target in PCa via disrupting the PCAT7‐miR‐324‐5p‐TGF‐β loop.

## Conflict of interest

The authors declare no conflict of interest.

## Author contributions

XP and DR developed ideas and drafted the manuscript. CL, YD, and ZW conducted the experiments and contributed to the analysis of data. XZ, SH, and QY contributed to the analysis of data. YL and HD contributed to the analysis of data and revised the manuscript. WG edited the manuscript. All authors contributed to revise the manuscript and approved the final version for publication.

## Supporting information


**Fig. S1.** Identification of PCAT7 as a pro‐bone metastasis‐relevant lncRNA in PCa.

**Fig. S2.** PCAT7 promotes bone metastasis of PCa cells.

**Fig. S3.** PCAT7 acts as a competitive endogenous RNA for miR‐324‐5p in PCa.

**Fig. S4.** PCAT7 promotes bone metastasis by activating TGF‐β signaling via sponging miR‐324‐5p.

**Fig. S5.** PCAT7 disrupts miR‐324‐5p‐mediated suppression on TGFBR1.

**Fig. S6.** SP1 promotes PCAT7 transcription in PCa cells.

**Fig. S7.** Clinical relevance of PCAT7‐miR‐324‐5p‐TGF‐β signaling in PCa.

**Table S1.** List of primers used for real‐time RT‐PCR.

**Table S2.** Clinicopathological features of 57 prostate cancer patients.

**Table S3.** List of primers used for ChIP assay.

**Table S4.** Relationship between PCAT7 and clinicopathological features in 57 patients with prostate cancer.
