# Supplementary figures and images for: SMAD3/SP1 complex‐mediated constitutive active loop between lncRNA PCAT7 and TGF‐β signaling promotes prostate cancer bone metastasis
Source: Mol Oncol. 2020 Feb 8;14(4):808–28. doi: 10.1002/1878-0261.12634 (PMC7138406; doi:10.1002/1878-0261.12634)

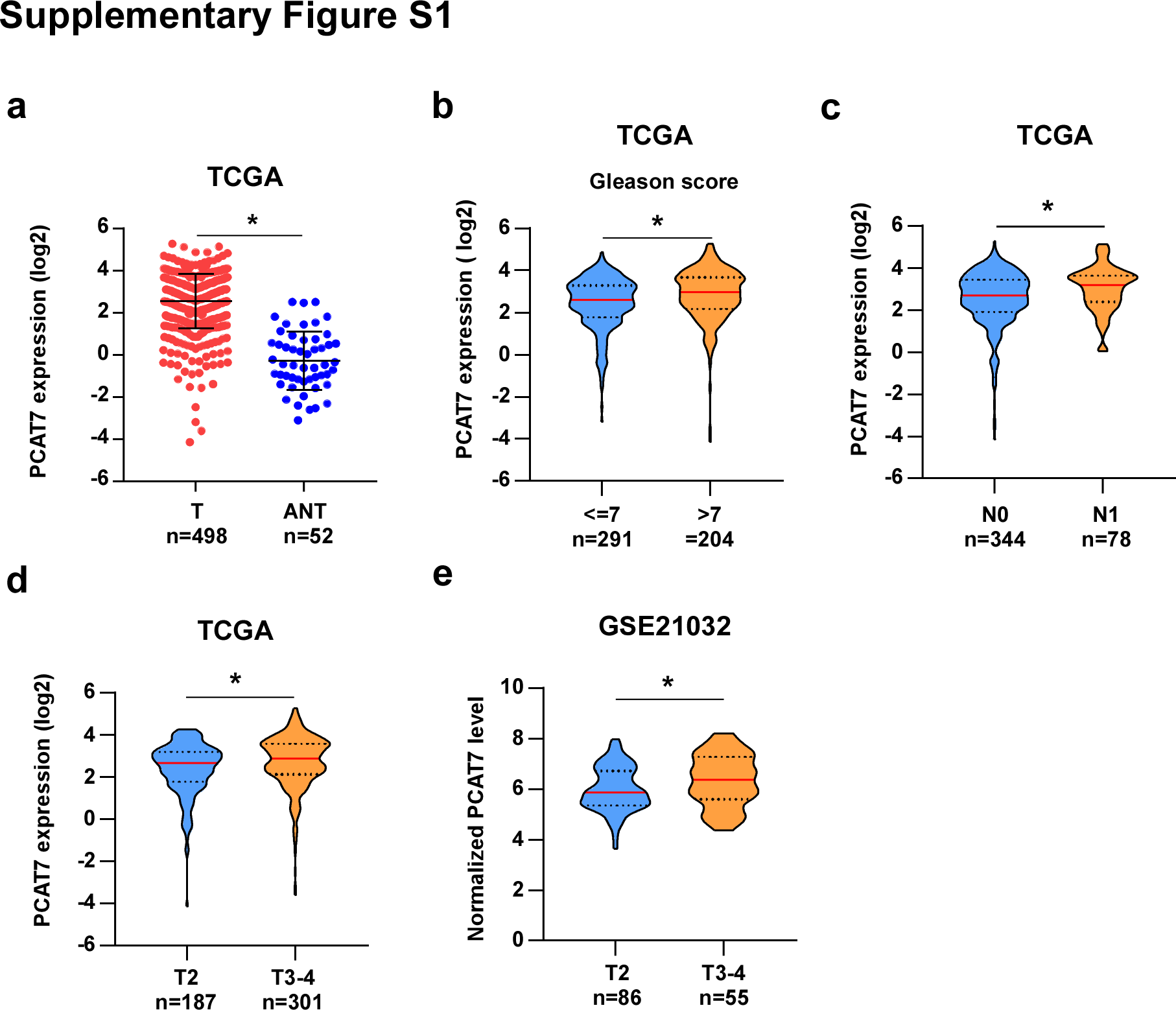

Supplement: Supplementary file 1 — Fig. S1. Identification of PCAT7 as a pro‐bone metastasis‐relevant lncRNA in PCa. [file MOL2-14-808-s001.tif]

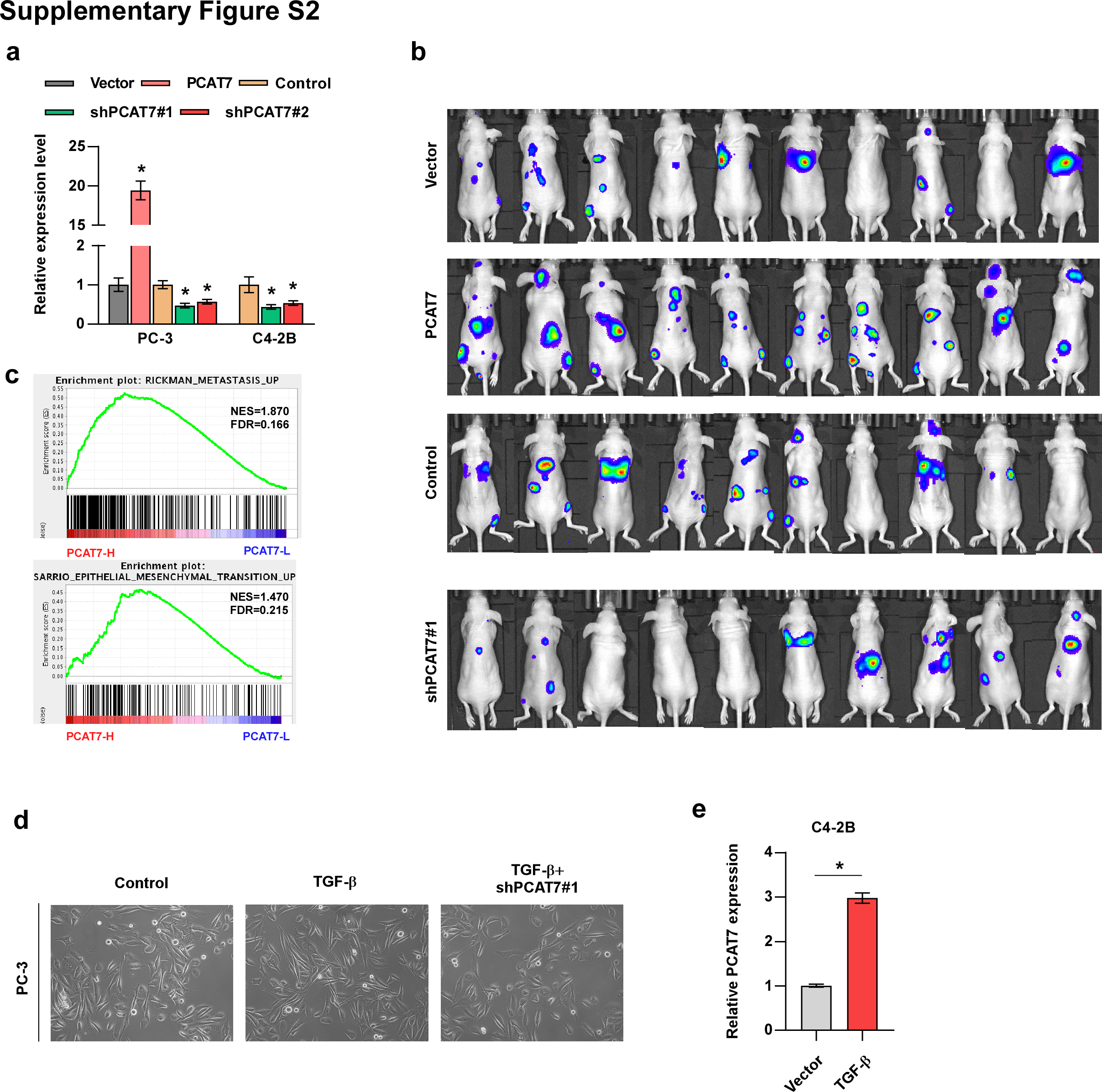

Supplement: Supplementary file 2 — Fig. S2. PCAT7 promotes bone metastasis of PCa cells. [file MOL2-14-808-s002.tif]

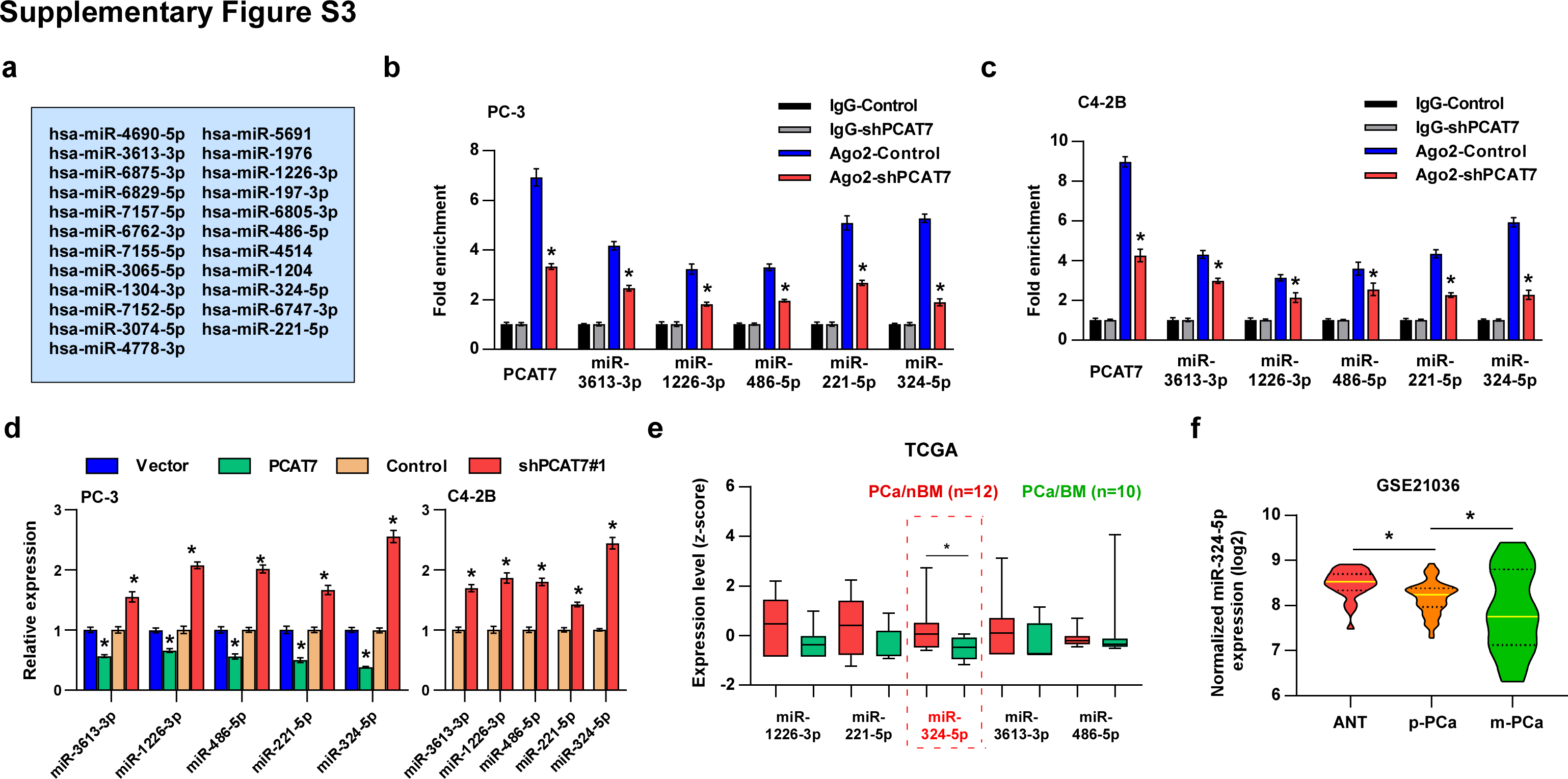

Supplement: Supplementary file 3 — Fig. S3. PCAT7 acts as a competitive endogenous RNA for miR‐324‐5p in PCa. [file MOL2-14-808-s003.tif]

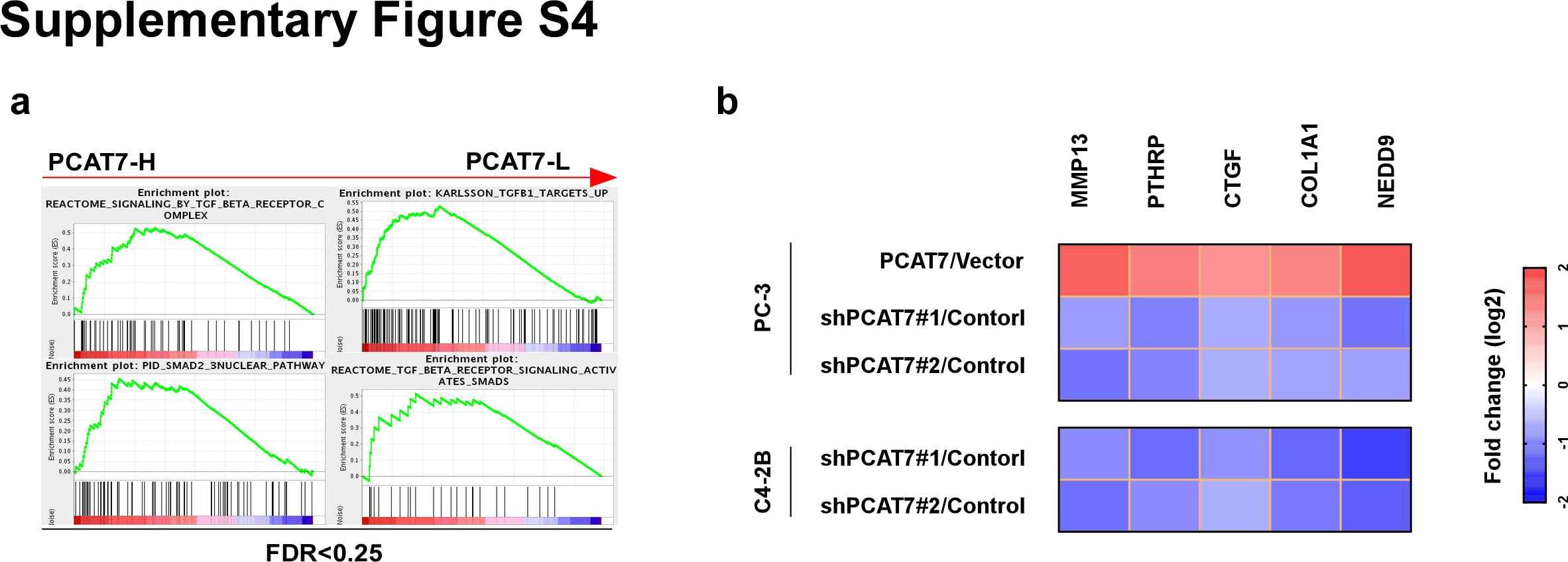

Supplement: Supplementary file 4 — Fig. S4. PCAT7 promotes bone metastasis by activating TGF‐β signaling via sponging miR‐324‐5p. [file MOL2-14-808-s004.tif]

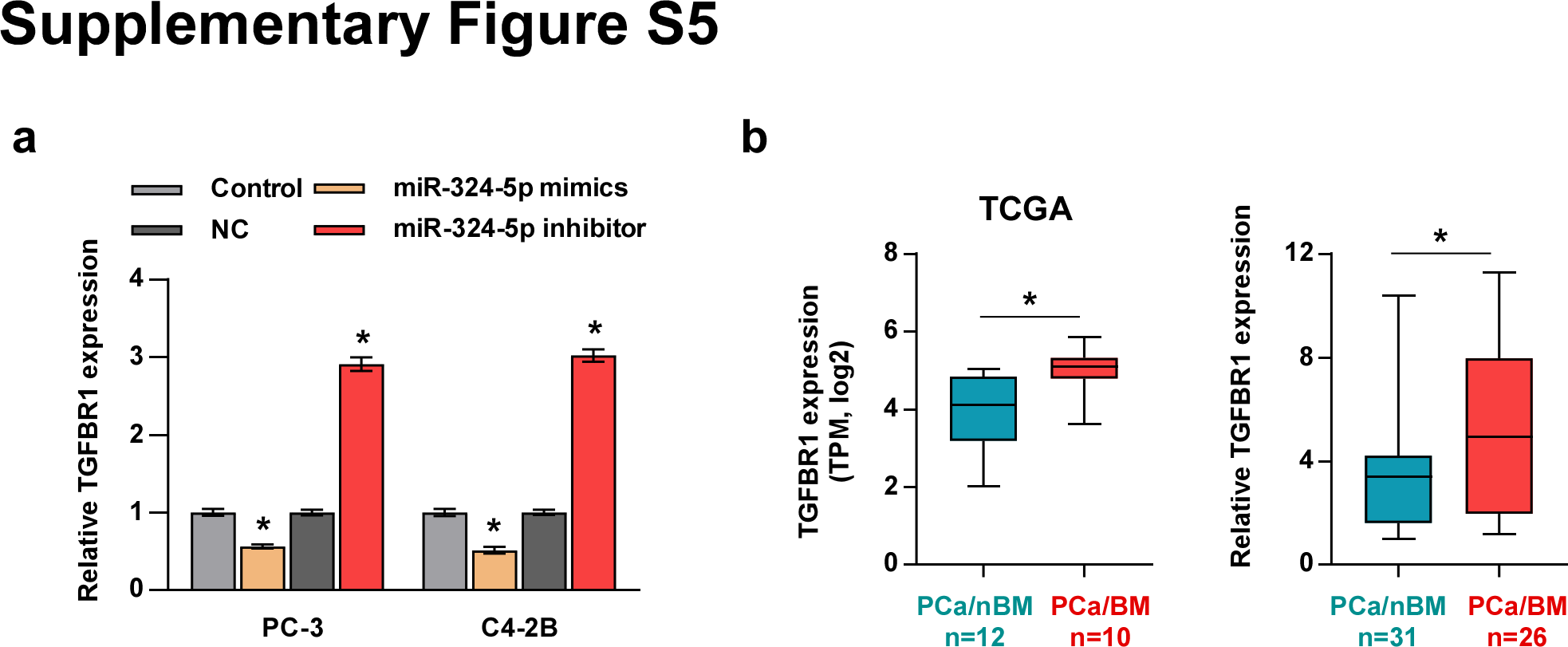

Supplement: Supplementary file 5 — Fig. S5. PCAT7 disrupts miR‐324‐5p‐mediated suppression on TGFBR1. [file MOL2-14-808-s005.tif]

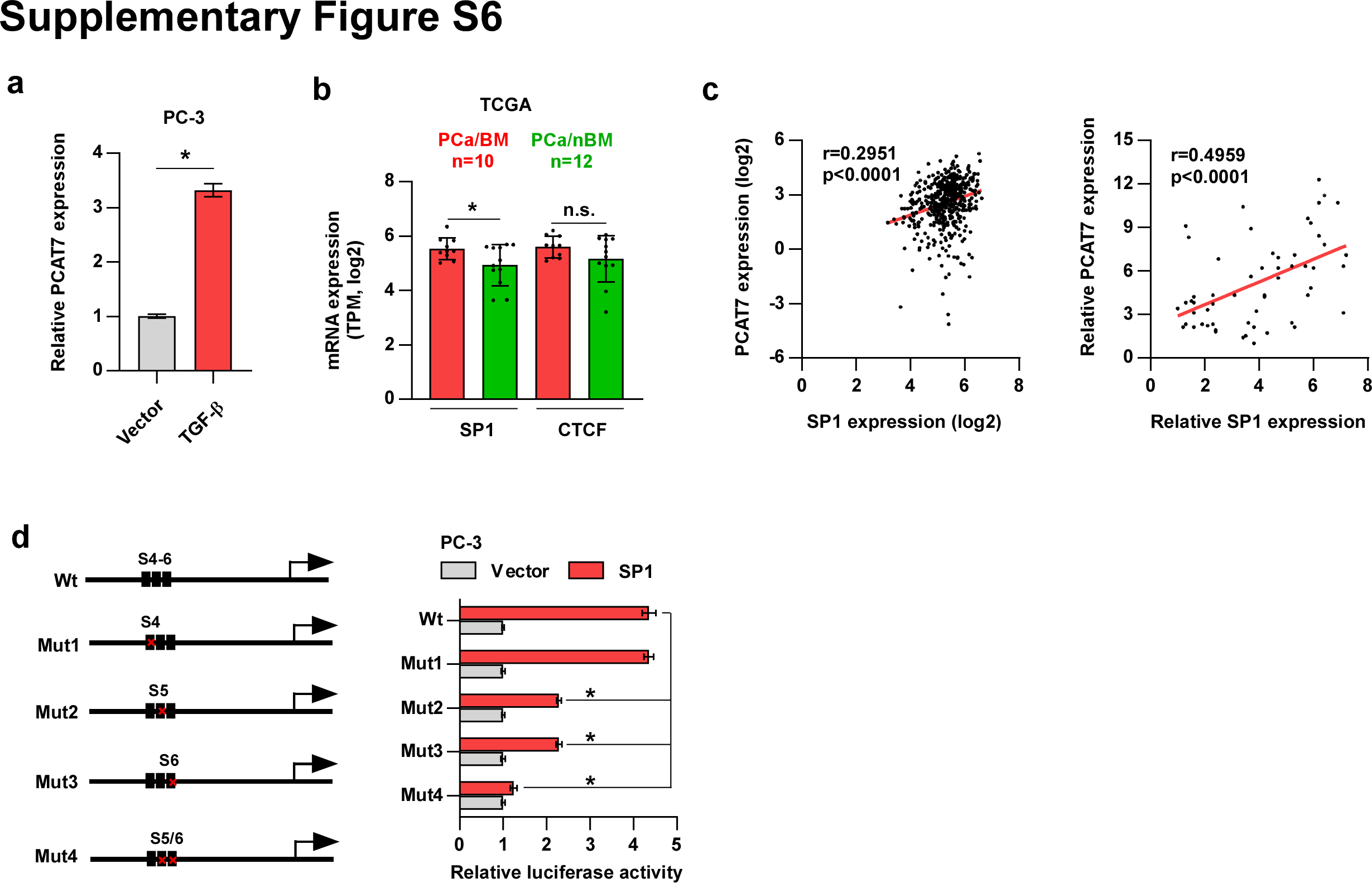

Supplement: Supplementary file 6 — Fig. S6. SP1 promotes PCAT7 transcription in PCa cells. [file MOL2-14-808-s006.tif]

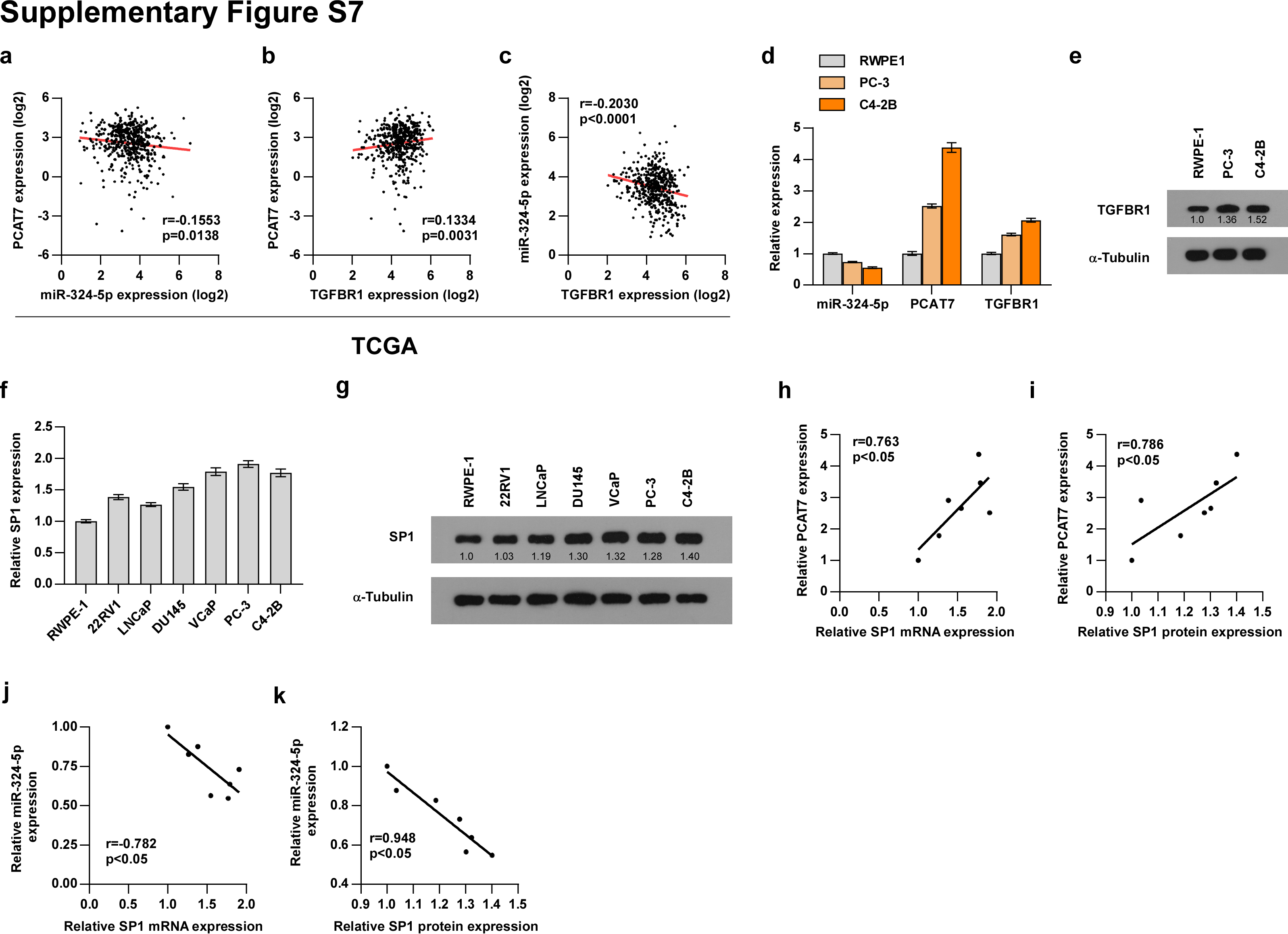

Supplement: Supplementary file 7 — Fig. S7. Clinical relevance of PCAT7‐miR‐324‐5p‐TGF‐β signaling in PCa. [file MOL2-14-808-s007.tif]
