# Supplementary material for: SMAD3/SP1 complex‐mediated constitutive active loop between lncRNA PCAT7 and TGF‐β signaling promotes prostate cancer bone metastasis
Source: Mol Oncol. 2020 Feb 8;14(4):808–28. doi: 10.1002/1878-0261.12634 (PMC7138406; doi:10.1002/1878-0261.12634)
Supplement: Supplementary file 8 — Table S1. List of primers used for real‐time RT‐PCR. [file MOL2-14-808-s008.docx]

**Table S1. List of primers used for real-time RT-PCR.**

| **Primer** | |
| --- | --- |
| PCAT7-up | CTTGGCTGTGGTCACTCTGA |
| PCAT7-dn | ACACACAGTTGGGTTCACCA |
| SP1-up | ACGCTTCACACGTTCGGATGAG |
| SP1-dn | TGACAGGTGGTCACTCCTCATG |
| TGFBR1-up | GACAACGTCAGGTTCTGGCTCA |
| TGFBR1-dn | CCGCCACTTTCCTCTCCAAACT |
| PTHRP-up | ACTCGCTCTGCCTGGTTAGA |
| PTHRP-dn | GGAGGTGTCAGACAGGTGGT |
| CTGF-up | GCTACCACATTTCCTACCTAGAAATCA |
| CTGF-dn | GACAGTCCGTCAAAACAGATTGTT |
| NEDD9 -up | CTACAGGGTAAGGAGGAGTTT |
| NEDD9-dn | TGGGTCTCACATTGGTCAT |
| MMP13-up | AACATCCAAAAACGCCAGAC |
| MMP13-dn | GGAAGTTCTGGCCAAAATGA |
| COL1A1-up | CCTGGATGCCATCAAAGTCT |
| COL1A1-dn | CGCCATACTCGAACTGGAAT |
| VEGFA-up | AAGGAGGAGGGCAGAATCAT |
| VEGFA-dn | CACACAGGATGGCTTGAAGA |
| GAPDH-up | ATTCCACCCATGGCAAATTC |
| GAPDH-dn | TGGGATTTCCATTGATGACAAG |
