# Supplementary material for: SMAD3/SP1 complex‐mediated constitutive active loop between lncRNA PCAT7 and TGF‐β signaling promotes prostate cancer bone metastasis
Source: Mol Oncol. 2020 Feb 8;14(4):808–28. doi: 10.1002/1878-0261.12634 (PMC7138406; doi:10.1002/1878-0261.12634)
Supplement: Supplementary file 9 — Table S2. Clinicopathological features of 57 prostate cancer patients. [file MOL2-14-808-s009.docx]

**Table S2.** **Clinicopathological features of 57 prostate cancer patients**

| Parameters | Number of cases |
| --- | --- |
| Age (years) |  |
| <71 | 28 |
| ≥71 | 29 |
| Median | 71 |
| Differentiation |  |
| Well/moderate | 25 |
| Poor | 32 |
| Serum PSA at diagnosis, µg/ml |  |
| <65.2 | 28 |
| ≥65.2 | 29 |
| Median | 65.2 |
| SD | 514.3 |
| Mean | 213.2 |
| Gleason grade |  |
| ≤7 | 33 |
| >7 | 24 |
| PCAT7 expression |  |
| <4.3 | 27 |
| ≥4.3 | 30 |
| Median | 4.3 |
| SD | 3.0 |
| Mean | 5.2 |
| BM status |  |
| nBM | 31 |
| BM | 26 |

**Abbreviation: PSA, Prostate-specific Antigen; SD, Standard Deviation; BM, Bone Metastasis; n-BM: Non-bone Metastasis.**
