# Supplementary material for: SMAD3/SP1 complex‐mediated constitutive active loop between lncRNA PCAT7 and TGF‐β signaling promotes prostate cancer bone metastasis
Source: Mol Oncol. 2020 Feb 8;14(4):808–28. doi: 10.1002/1878-0261.12634 (PMC7138406; doi:10.1002/1878-0261.12634)
Supplement: Supplementary file 10 — Table S3. List of primers used for ChIP assay. [file MOL2-14-808-s010.docx]

**Table S3. List of primers used for ChIP assay**

| **Primer** | |
| --- | --- |
| P1-F | TCATGAGGCAAGCTGCAGAG |
| P1-R | GCCAGCGTGACAAGGTGAAG |
| P2-F | CGCGTAGGAGAAACTCAGAC |
| P2-R | ACGCTCTGTGTTGTGGAGAC |
| P3-F | GCAGTGTTAGCACCCGATAG |
| P3-R | TGGTTTAAGGGCGTGGTCCG |
