# Supplementary material for: SMAD3/SP1 complex‐mediated constitutive active loop between lncRNA PCAT7 and TGF‐β signaling promotes prostate cancer bone metastasis
Source: Mol Oncol. 2020 Feb 8;14(4):808–28. doi: 10.1002/1878-0261.12634 (PMC7138406; doi:10.1002/1878-0261.12634)
Supplement: Supplementary file 11 — Table S4. Relationship between PCAT7 and clinicopathological features in 57 patients with prostate cancer. [file MOL2-14-808-s011.docx]

**Table S4. Relationship between PCAT7 and clinicopathological features in 57 patients with prostate cancer.**

| Parameters | Number of cases | PCAT7 expression | | P-values |
| --- | --- | --- | --- | --- |
|  |  | Low | High |  |
| Age (years) |  |  |  |  |
| <71 | 28 | 14 | 14 |  |
| ≥71 | 29 | 13 | 16 | 0.346 |
| Differentiation |  |  |  |  |
| Well/moderate | 25 | 16 | 9 |  |
| Poor | 32 | 11 | 21 | <0.05* |
| Serum PSA |  |  |  |  |
| <65.2 | 28 | 20 | 8 | <0.05* |
| ≥65.2 | 29 | 7 | 22 |  |
| Gleason grade |  |  |  |  |
| ≤7 | 24 | 17 | 7 |  |
| >7 | 33 | 10 | 23 | <0.05* |
| BM status |  |  |  |  |
| nBM | 31 | 20 | 11 |  |
| BM | 26 | 7 | 19 | <0.05* |
