# Supplementary material for: SMAD3/SP1 complex‐mediated constitutive active loop between lncRNA PCAT7 and TGF‐β signaling promotes prostate cancer bone metastasis
Source: Mol Oncol. 2020 Feb 8;14(4):808–28. doi: 10.1002/1878-0261.12634 (PMC7138406; doi:10.1002/1878-0261.12634)
Supplement: Supplementary file 12 [file MOL2-14-808-s012.docx]

**Figure Legends**

**Figure S1. Identification of PCAT7 as a pro-bone metastasis-relevant lncRNA in PCa**

**(a)** PCAT7 expression in PCa tissues (n=498) and adjacent normal tissues (n=52) in TCGA dataset. **P* < 0.05 **(b)** PCAT7 expression in PCa tissues with different Gleason score in TCGA dataset. **P* < 0.05 **(c)** PCAT7 expression in PCa tissues with different lymph node metastasis status in TCGA dataset. **P* < 0.05 **(d)** PCAT7 expression in PCa tissues with different tumor volume in TCGA dataset. **P* < 0.05 **(e)** PCAT7 expression in PCa tissues with different tumor volume in GEO dataset (GSE21032). **P* < 0.05

**Figure S2. PCAT7 promotes bone metastasis of PCa cells.**

**(a**) PCAT7 expression in the indicated PCa cells. **(b**) Gene set enrichment analysis (GSEA) revealed that PCAT7 expression significantly and positively correlated with the metastasis and EMT-related gene signature. **(c**)TGF-β upregulated PCAT7 expression in C4-2B cells. **P* < 0.05

**Figure S3. PCAT7 acts as a competitive endogenous RNA for miR-324-5p in PCa**

**(a**) Twenty-three miRNAs which may bind to PCAT7 predicted by lncBASE and lncRNASNP2. (**b** and **c)** Ago2 RNA immunoprecipitation (RIP) assay for the amount of PCAT7, miR-3613-3p, miR-1226-3p, miR-485-5p, miR-221-5p and miR-324-5p in the indicated groups. **P* < 0.05 **(d**) Real-time PCR analysis of miRNAs expression in the indicated groups. **P* < 0.05 **(e**) The expression of five miRNAs in PCa/nBM (n=11) and PCa/BM(n=9) in TCGA dataset. **P* < 0.05 **(f**) miR-324-5P expression in adjacent normal tissues (ANT, n=29), primary PCa tissues (p-PCa, n=131) and metastatic PCa tissues (m-PCa, n=19) in GEO dateset (GSE21032). **P* < 0.05

**Figure S4. PCAT7 promotes bone metastasis by activating TGF-β signaling via sponging miR-324-5p**

**(a**) Gene set enrichment analysis (GSEA) revealed that PCAT7 expression significantly and positively correlated with TGF-β pathway. **(b**) Real-time PCR analysis of downstream bone metastasis-related genes of the TGF-β pathway, including CTGF, PTHRP, NEDD9, MMP13, COL1A1 and VEGFA in the indicated groups.

**Figure S5. PCAT7 disrupts miR-324-5p-mediated suppression on TGFBR1**

**(a)** Real-time PCR analysis of TGFBR1 expression in PCa cells treated with miR-324-5p mimics or inhibitor. **P* < 0.05 **(b**) The expression of TGFBR1 in PCa/nBM (n=12) and PCa/BM(n=10) in TCGA dataset (left panel). The expression of TGFBR1 in PCa/nBM (n=31) and PCa/BM(n=26) in our clinical samples (right panel). **P* < 0.05

**Figure S6. SP1 promotes PCAT7 transcription in PCa cells.**

**(a**) TGF-β upregulated PCAT7 expression in PC-3 cells. **P* < 0.05 **(b**) SP1 and CTCF expression in PCa/nBM (n=12) and PCa/BM(n=10) in TCGA dataset. **P* < 0.05 **(c**) The positive correlation between SP1 and PCAT7 in TCGA dataset and our PCa patients. **(d**) The designed different mutations in PCAT7 promotor (left panel). The effect of these mutations on the luciferase activity of PCAT7 promotor (right panel). **P* < 0.05

**Figure S7. Clinical relevance of PCAT7-miR-324-5p-TGF-β signaling in PCa**

**(a**) PCAT7 expression was negatively correlated with miR-324-5p expression in TCGA dataset. **(b**) PCAT7 expression was positively correlated with TGFBR1 expression in TCGA dataset. **(c**) miR-324-5p expression was negatively correlated with TGFBR1 expression in TCGA dataset.
